# Patient‐specific quality assurance at the Heidelberg Ion Beam Therapy Center: 10 years experience in treatment plan verification

**DOI:** 10.1002/mp.70237

**Published:** 2025-12-26

**Authors:** Abdallah Qubala, Christian P. Karger, Julian Horn, Marcus Winter, Malte Ellerbrock, Oliver Jäkel, Katrin Henkner

**Affiliations:** ^1^ Heidelberg Ion Beam Therapy Center (HIT) Department of Radiation Oncology Heidelberg University Hospital Heidelberg Germany; ^2^ National Center for Radiation Research in Oncology (NCRO) Heidelberg Institute for Radiation Oncology (HIRO) Heidelberg Germany; ^3^ Division of Medical Physics in Radiation Oncology German Cancer Research Center (DKFZ) Heidelberg Germany; ^4^ Faculty of Medicine University of Heidelberg Heidelberg Germany

**Keywords:** dose measurements, dose verification, helium and carbon ion beam therapy, particle therapy, patient specific quality assurance, proton, spot scanning, water phantom

## Abstract

**Background:**

To ensure accurate, safe, and reproducible patient treatments, it is essential to have precise knowledge and a solid understanding of patient‐specific quality assurance (PSQA). For many years, the delivery of doses to all patients has been verified using dosimetric measurements. However, these measurements require substantial work, and the reasons for the occasional deviations are unclear. For these reasons, alternative methods such as independent dose calculations (IDCs) and analysis of beam‐monitor log files are increasingly discussed in the particle therapy community. Nevertheless, before replacing dose‐verification measurements with other methods, existing measurement data should be thoroughly analyzed to determine what can be learned from them and how they compare with potential alternatives. These alternative methods are mentioned in this work only to provide context and to outline possible directions for future studies.

**Purpose:**

To evaluate the dosimetric accuracy and efficiency of PSQA using a water phantom (WP) over a 10‐year period at the Heidelberg Ion Beam Therapy Center (HIT).

**Methods:**

Between 2016 and 2025, 23014 treatment fields with protons, carbon, or helium ions were verified using a WP equipped with 24 pinpoint ionization chambers. The patient treatment plans were recalculated in the water phantom geometry and compared to measured absolute doses. The data were categorized by treatment room, ion species, treatment planning systems (TPS), range shifter (RaShi) use, indication, depth, and target volume, excluding measurements with human errors. Statistical analysis compared measured and calculated doses, focusing on mean, maximum, and minimum dose deviations. Furthermore, the workflow efficiency was assessed based on the beam time required for dosimetric verification, as well as the total time needed for preparation and analysis.

**Results:**

Mean dose deviations were in general slightly negative (*t*‐test, *p* < 0.01), within ±1 % across all categories (total mean ± SD  =  –0.50 ± 0.90 %), with 91 % of fields passing institutional ±5 % tolerances. Further, significant differences (*p* < 0.01) were also observed between treatment rooms, ion species, TPS platforms, and RaShi settings. Additionally, the RayStation TPS showed lower deviations than the Syngo TPS, and helium ions had the smallest deviations. Moreover, repeated verifications reduced variability but without significant improvement. Correlations with target depth or volume were statistically significant but clinically negligible. Less than 1 % of maximum and minimum dose measurements exceeded ±7 % annually. Finally, over 4308 h of beam time, preparation, and analysis were spent on PSQA during the 10‐year period.

**Conclusions:**

PSQA at HIT demonstrated high dosimetric accuracy and delivery stability. Integration of IDCs and log file analysis may improve efficiency and allow to omit verification measurements in well‐established cases without compromising patient safety and treatment quality, if the extensive machine QA program is maintained.

## 1 INTRODUCTION

The primary goal of radiotherapy is to deliver high dose to the tumor while sparing adjacent normal tissues. Compared to photon beams, proton and ion beams exhibit a superior depth dose profile, termed as spread‐out Bragg‐peak (SOBP).^1^ Combined with pencil beam scanning,^2^, ^3^ highly conformal dose distributions to the tumor can be delivered using only a few beam directions (treatment fields), leading to a significantly lower integral dose to organs at risk (OAR).^4^ Heavier (e.g., carbon) ions additionally exhibit an increased relative biological effectiveness (RBE) in the tumor, which may further increase clinical efficacy.^4^, ^5^

Dose delivery using the dynamic pencil beam scanning requires a comprehensive quality assurance (QA) program, including regular constancy checks covering the entire treatment workflow.^6^, ^7^ However, due to the high cost of ion beam therapy, time‐efficient QA procedures are of particular importance.^8^ Verification of dose delivery for individual treatment plans prior to patient irradiation is part of the patient‐specific quality assurance (PSQA). However, there are no guidelines recommending specific PSQA methods to particle therapy.

Further, precise knowledge and good understanding of PSQA is necessary for accurate, safe, and reproducible patient treatments and good clinical results.^7^, ^9^ Over a long time, verification of dose delivery for all patients has been performed using dosimetric measurements.^3^, ^6^, ^10^, ^11^, ^12^, ^13^, ^14^, ^15^, ^16^ However, these measurements are time‐consuming and the underlying causes for occasionally observed deviations remain mostly unclear. For these reasons, alternative methods such as independent dose calculations (IDC)^17^, ^18^, ^19^, ^20^ and the analysis of log files of the beam monitor system^21^, ^22^, ^23^, ^24^ are discussed in particle therapy.^25^ For instance, a white paper on optimizing PSQA in particle therapy was prepared in March 2024 by the Particle Therapy Co‐Operative Group (PTCOG).^25^ However, before replacing dose verification measurements by other methods, existing measurements should be thoroughly analyzed to elucidate what can be learned from the result and how this compares with alternative methods. These methods are not evaluated in the present study and are referenced only to provide an overview of the current discussions and outline potential future directions for PSQA optimization in particle therapy. Comprehensive analyses of long‐term dosimetric results of patient‐specific QA in particle therapy are rare.^11^

At the Heidelberg Ion Beam Therapy Center (HIT), dose verification measurements have been executed since the start of clinical operation in 2009, using a commercialized version (PTW Freiburg, Germany) of a system consisting of a water phantom (WP) and a stack of 24 PinPoint ionization chambers (ICs).^13^ For this, the treatment fields were recalculated to the geometry of the WP by the treatment planning system (TPS) using the same algorithm as for patient dose calculation. Overall, this resulted in 9450 patients in 16 years of clinical operation at HIT.

The aim of this work is to evaluate the dose verification measurements performed for protons, carbon, and helium ions over the last 10 years at HIT. For this purpose, the quality of these dose verification measurements was assessed, considering several factors, such as treatment room, ion species, TPS, use of range shifter (RaShi), indication, depth, target volume, and passed or failed tolerances according to the tolerance limits. In addition, the irradiation time was recorded. Based on the results, a decision‐making strategy shall be developed within our clinical PSQA workflow (WF), supporting us in determining the most suitable risk‐adapted PSQA verification measure.

Finally, this work shall help to answer three important questions: (i) Shall dose verification measurements prior to the first irradiation be mandatory in ion beam therapy? (ii) Will omitting dosimetric verification measurements introduce additional risks? and (iii) What alternative procedures can be implemented to mitigate these risks and to ensure optimal and safe treatments?

## 2 MATERIALS AND METHODS

### 2.1 Beam delivery at HIT

HIT is a synchrotron‐based facility delivering protons, carbon, and helium ions for patient treatment.^26^ All patients are irradiated using an intensity and energy modulated raster scan technique.^2^, ^27^ Using two deflecting magnets, a pencil beam is scanned perpendicular to the beam direction across the tumor. The depth of the Bragg peaks is modulated by actively varying the energy of the ions. The dose is distributed over the tumor volume by superimposing Bragg peaks with different initial energies and lateral beam positions.

At each grid point, the number of particles and hence the dose can be controlled independently, thus delivering a highly conformal dose distribution to the target volume.^28^ For a typical target volume of 5 cm diameter with two irradiation fields, around 30 energy layers, 3500 spots and 2 × 10^9^ Helium particles are delivered to apply 2 Gy (RBE) per fraction. Treatment plans may be generated either by single‐field or multiple‐field optimization.

HIT operates three treatment rooms. Two rooms deliver the beam in fixed horizontal direction, and the third one uses an isocentric gantry with the ability to rotate around 360°. Proton, carbon, and helium (since 2021)^29^ ions can be delivered at HIT. Indications such as craniospinal irradiation, focal irradiation of head and neck or brain tumors, lymphoma, prostate cancer, liver, pancreas, skull base, and spinal sarcoma are treated.

### 2.2 PSQA at HIT

**FIGURE 1:**
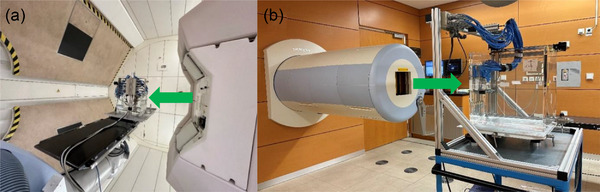
Setup for dose verification measurement employing a WP with 24 PinPoint‐ICs readouts by 2 12‐channel electrometers.^13^ (a) WP positioned on the treatment table at the gantry treatment room with the gantry positioned at 90°. (b) WP positioned at the horizontal treatment room. In both cases, the beam enters the WP perpendicular (green arrows) to the WP surface. IC = ionization chamber, WP = water phantom.

**FIGURE 2:**
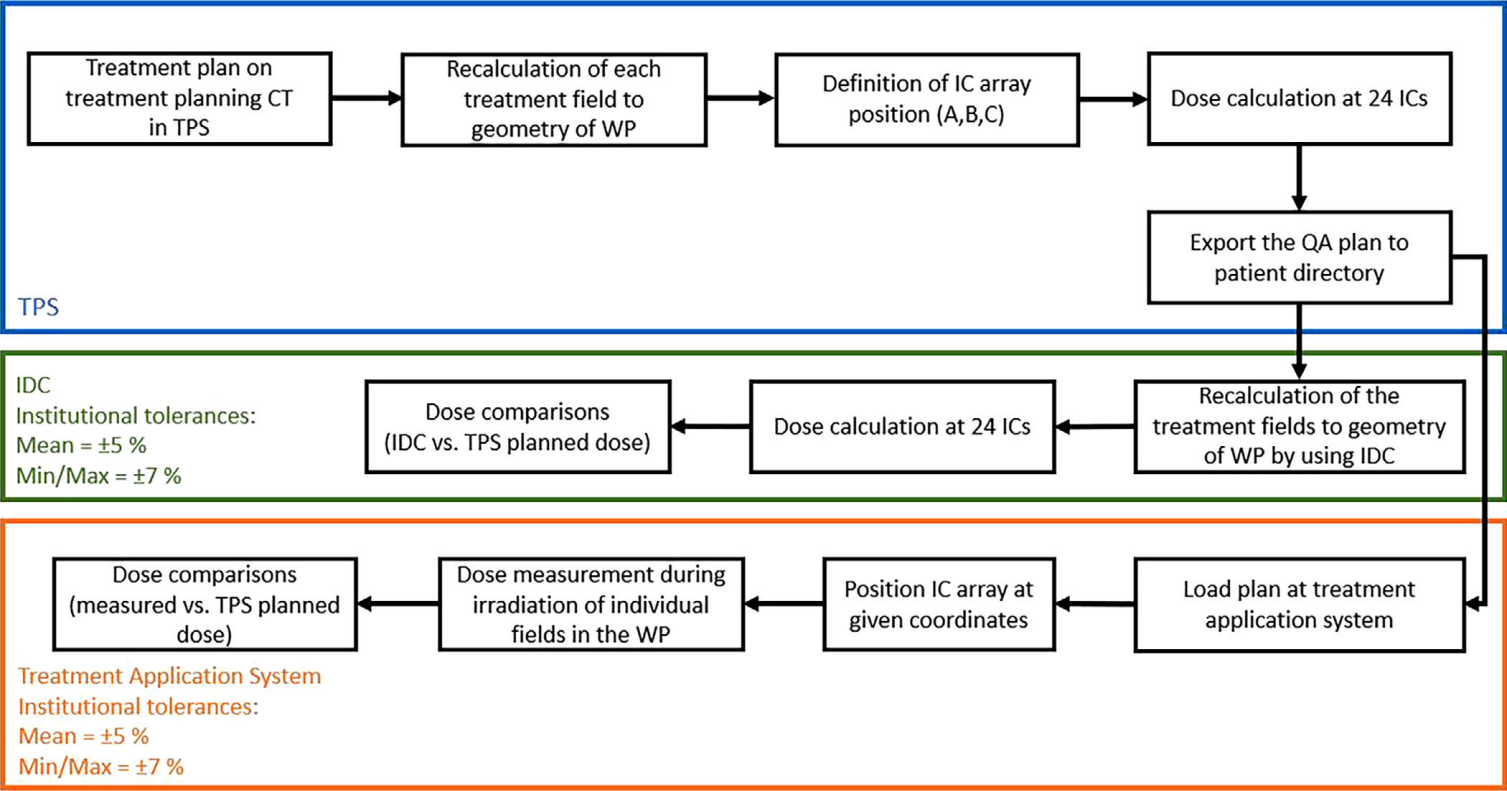
Current WF of PSQA by both IDC and dosimetric measurement at HIT. The blue box shows the steps of preparing the QA plan in TPS. The green box presents the IDC WF, and the orange one shows the WF of the dosimetric verification in the WP. A, longitudinal axis of the WP (depth); B, vertical axis; C, lateral axis; CT, computed tomography; IC, ionization chamber; IDC, independent dose calculation; QA, quality assurance; max, maximum; min, minimum,; TPS, treatment planning system; WP, water phantom.

At HIT, each single field of each patient is currently dosimetrically verified in the WP (Figure 1). In most cases, these measurements are performed before delivering the first fraction, however, the treatment plan may be temporally approved for a limited number of fractions by an IDC in the geometry of a WP. In these cases, dose verification measurements have to be completed at the latest by the tenth fraction or before 50 % of the planned fractions are delivered (Figure 2). The current IDC tool at HIT employs an in‐house pencil beam algorithm (PBA), which has been implemented independently from that of the treatment planning system. For offline‐adapted treatment plans that have initially been verified in the WP, no additional verification is required, provided that the adapted plan uses similar settings in the TPS as the original plan (Table 1).

**TABLE 1:** Tolerance limits for dose verification measurements and IDC together with the criteria for omitting verification measurements in case of plan adaptions at HIT.

PSQA approach	Institutional tolerances
WP measurement	Mean = ± 5% Minimum = −7 % Maximum = +7 %
IDC	Mean = ±5% Minimum = −7% Maximum = +7%
ATP	Same ion species Same fraction dose Same number of fields (contributing to a target volume) Comparable field contributions (e.g., energy layer spacing, spot spacing, grid size, and dose) Couch and gantry angles within about ±10° Same position of beam modifiers at similar positions: RiFi and RaShi (within about ±10 cm) Comparable energy ranges of the fields, energy range may be expanded by up to ∼20 energy levels. Comparable total particle number of all fields within about ±20 %: Individual fields may exhibit larger variations in particle number.

Abbreviations: ATP, adapted treatment plan; IDC, independent dose calculation; PSQA, patient‐specific quality assurance; RaShi, range shifter; RiFi, ripple filter; WP, water phantom.

**FIGURE 3:**
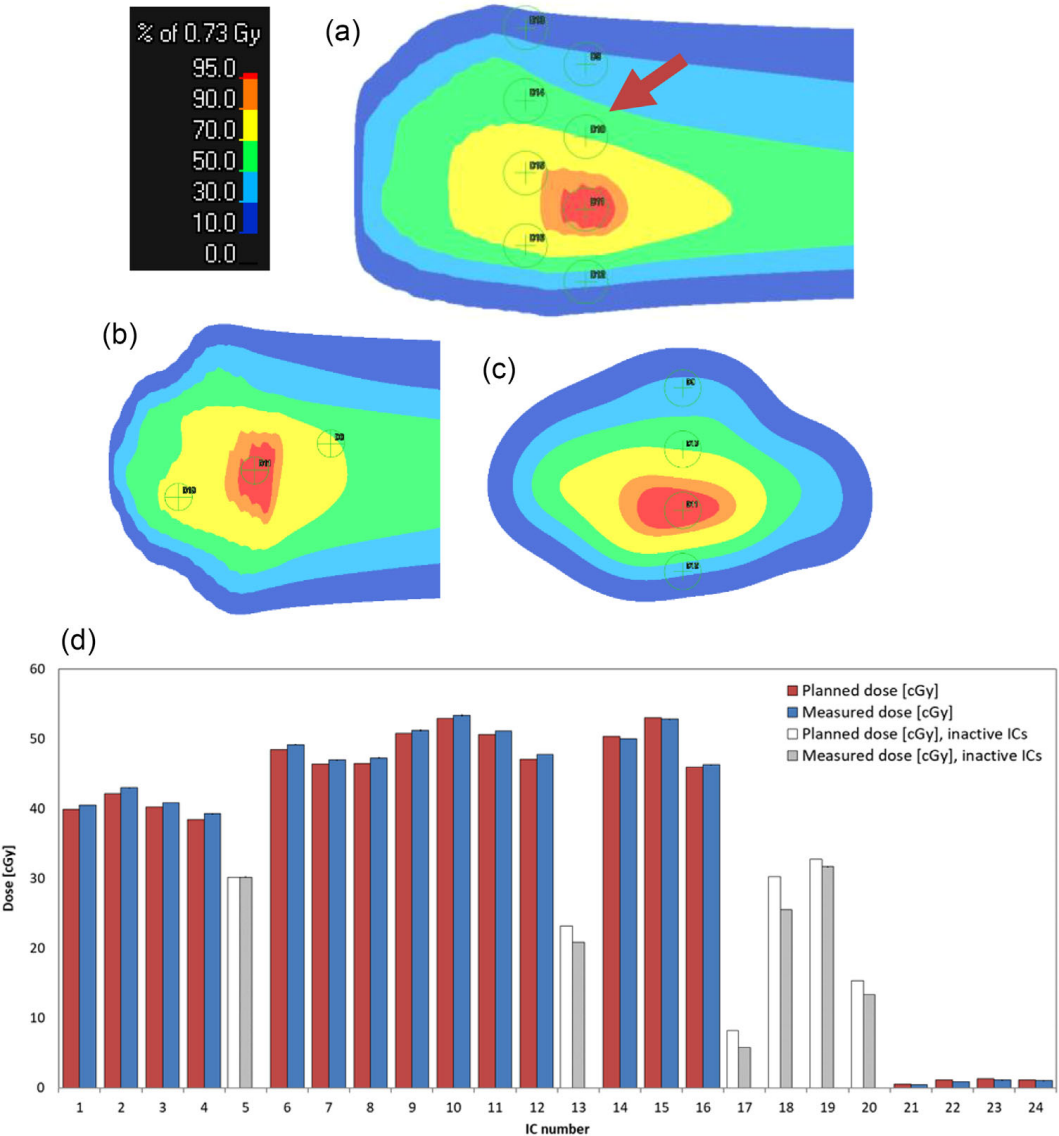
Example of a patient treatment plan verification delivered with a scanned helium ion beam at HIT. Measurements were performed in a WP using a stack of 24 pinpoint ICs marked by green circles with “+” in the coronal (a), transversal (b) and sagittal (c) view of the dose distribution in the RayStation TPS (RaySearch Laboratories, Stockholm, Sweden). The histogram displays the 24 calculated (red) and measured (blue) dose values at the IC positions. The grey bars denote ICs that were deactivated because the dose gradients were larger than 5% of the maximum planned dose. The dose gradients were estimated over the six neighboring voxels at the IC position in the TPS. IC, ionization chamber; TPS, treatment planning system, WP = water phantom.

In doing so, all plans at the gantry are recalculated for a horizontal beam (gantry at 90°), to be deliverable to the WP. The recalculated plan is referred to as the QA plan. After choosing the position of the IC stack, the 24 dose reference values at the IC positions are calculated at the effective point of measurement of the ICs (Figure 3). The QA plan for all fields is then exported to the treatment machine, and after the IC stack is setup to the planned position, the fields are delivered to the WP (MP3‐P, PTW Freiburg, Germany) equipped with 24 PinPoint ICs (Type 31015,^30^ PTW Freiburg, Germany) read out by two 12 channel‐electrometers (Multidos, PTW Freiburg, Germany).^13^ Before measuring patient‐specific treatment plans, the beam monitor calibration was checked and corrected, if necessary, within daily QA.

The IC stack consists of 6 rows of 4 ICs each, positioned without overlap on three different heights. During setup, a marked reference point of the IC stack is aligned to a reference point of the water phantom, and the individual chamber positions are defined with respect to the reference point of the IC stack. These IC positions are displayed in the dose distribution by the TPS. A comprehensive description of the alignment and positioning procedure of the IC stack relative to the WP is provided in^13^. IC's are calibrated in dose to water and absolute doses are measured according to TRS398.^28^ This approach is also recommended in the latest Code of Practice of the International Atomic Energy Agency, Technical Report Series TRS‐398.^31^ As a preparation for the measurement, the patient treatment plan is recalculated to the geometry of the WP using the dose calculation algorithm of the TPS (Figure 2).

Note that only the air density correction and a beam quality correction factor of 1.026 were considered for the absolute dosimetry for all ion species at our facility.^29^ The beam enters the WP under 90° (Figure 1) and typically one measurement per field is performed, covering the region of high doses and/or the distal fall‐off. Figure 2 summarizes the workflow for PSQA at HIT.

After the irradiation, the measured dose values are imported into the TPS for comparison with calculations. Tolerance limits are based on mean deviation (mean) of measured and calculated dose normalized to the maximum dose of the field as well as the minimum and maximum deviation (Table 1, WP measurements). In the case of a failed verification measurement, the measurement was typically repeated. If the repeated measurement still did not meet tolerance criteria and the deviation could not be otherwise explained or justified, the treatment plan was modified to ensure compliance with the defined tolerances before clinical delivery.

In the case of IDC, the 24 dose values at the IC positions are calculated by our in‐house developed PBA and compared with doses at the 24 chamber positions of the QA plan. For acceptance, same tolerances limits are used (Table 1, IDC). In case of plan adaptations, dose verification measurements are omitted, if specific criteria apply (Table 1, ATP). As further part of PSQA, data integrity after transferring the treatment plan from the TPS to the treatment machine is checked by our in‐house tool. Among others, the following tags are checked: approval status, DICOM plan label, review date and time, patient name, patient ID, field name, number of scan spots in DICOM plan and in RayStation TPS, particle number distribution (position, focus, and energy), beam modifiers, their positions and isocenter positions of treatment fields.

### 2.3 Treatment planning and field characteristics

Two different TPS were used for dose calculation during the reported period: (i) Syngo RT planning TPS VC13A, VC13B, and VC13C (Siemens Healthineers, Erlangen, Germany) and (ii) RayStation TPS 11B and 2024B (in use since 2025). Syngo used the PBA for protons and carbon ions while RayStation uses the PBA for carbon and helium ions and Monte Carlo (MC) calculations for protons. Since 2023, only the RayStation TPS is used for patients at HIT. For Syngo, no algorithmic changes relevant to dose calculation were identified across versions. For RayStation, the upgrade from 11B to 2024B resulted in negligible differences, within ±1 % for the mean dose across different depths, and no effect was seen in the PSQA plans analyzed in this study.

For verification planning, an isotropic dose grid resolution of either 1 or 2 mm was selected for RayStation TPS while 2 mm was used for Syngo TPS. The minimum full width at half maximum values of the pencil beam were 6 or 10 mm for carbon ions, 6 mm for helium ions and 8 mm for protons.

The planning target volumes considered in this work ranged between 10 and 10000 cm^3^ (sacral chordoma), and the water‐equivalent target depth ranged from 0 to 250 mm. Figure 3 displays an example of a QA plan calculated by the RayStation TPS.

### 2.4 Data collection and statistical analysis

This analysis encompasses dose measurements in the WP of 23014 treatment fields performed between January 2016 and June 2025. Measurements included the horizontal and gantry treatment rooms and all ion species. The measured data were categorized into different groups (treatment room, ion species, TPS, use of RaShi, indication, depth, target volume, and passed or failed tolerances according to the tolerance limits in Table 1). Measurements with retrospectively identified human operating errors were excluded from the analysis. This included incorrect positioning or operation of the WP, wrong calculated air gaps between the WP and nozzle, incorrect setting of the WP limits or use of wrong calibration factors of the ICs. Human operating errors are mostly detected and corrected directly after the measurement and documented in QA protocols. During analysis, these protocols were checked and the respective measurements were excluded from the analysis.

Statistical analysis is based on the field‐specific comparison of measured and calculated doses expressed as mean deviation (mean), the respective standard deviation (SD) as well as the minimum and maximum deviations. Deviations are normalized to the maximum dose of the measured field ((measurement dose—TPS dose)/*D*_max_). The maximum dose (*D*_max_) refers to the entire treatment field in the TPS, ensuring a consistent normalization across all measurement positions. These parameters do not include measurement positions from excluded (deactivated) chamber positions, where the calculated dose gradients in mGy/mm is greater than 5 % of the maximum dose, indicating very steep dose gradients. These dose gradients were estimated over the six neighboring voxels at the IC position in the TPS. This method was applied consistently for both Syngo and RayStation, using TPS‐provided gradient values.

Based on the field‐specific mean deviations of all measurements, the total mean deviation and its respective SD were determined for each group analyzed in this work. Further, the total maximum and minimum deviations of all minima and maxima were determined. In this work, all tables and figures are based on these parameters, if not otherwise stated.

Normality of the dosimetric parameters (mean, minimum and maximum deviations) were tested by the Shapiro–Wilk test. Because of its robustness for large (*n* ≥ 30) and symmetrically distributed sample sizes, even in case of deviations from normality, the one‐sample *t*‐test was used to test the total mean deviation for systematic shifts against zero. Since the distributions of the minimum and maximum deviations were strongly asymmetric, the one‐sample Wilcoxon signed‐rank test was applied instead.

In addition, the total mean deviations between categories (treatment room, ion species, TPS, use of RaShi, indication, depth, target volume, and repeated measurements) were evaluated. For two‐group comparisons, Levene's test determined whether a two‐sample *t*‐test (equal variances) or Welch's *t*‐test (unequal variances) was applied. For comparison of more than two groups, a one‐way ANOVA test was conducted followed by Tukey's HSD post hoc test in case of significance. Correlations were evaluated using the Pearson correlation coefficient (r). Additionally, the time required for dosimetric verification was recorded to assess the PSQA workflow efficiency. Results were considered significant if *p* value < 0.01. All statistical analyses and plots were performed in MATLAB 2022a (MathWorks, Natick, MA).

## 3 RESULTS

### 3.1 Measurement characteristics

Table 2 shows the characteristics of the analyzed measurement, and Table 3 summarizes the measurements for the last 10 years of clinical operation. In total, 23014 ion fields have been measured in the WP for different indications using different ion species (^1^H, ^12^C, and ^4^He), treatment rooms (Hx, Ga), RaShi settings, and TPS (RayStation, Syngo). Results are separately displayed for these categories and for combinations of treatment rooms and ion species in Table 2. Additionally, Table 3 shows that more measurements were performed at the horizontal than at the gantry treatment rooms, and the number of proton fields was slightly higher than for carbon ions. Moreover, Table 3 shows that 75 % of the performed measurements were applied without using the RaShi.

**TABLE 2:** Summary of all measurements. Measurements were additionally separated according to treatment room, ion species, RaShi setting and indication as well as combinations of ion species and treatment room. The mean, SD, minimum, and maximum values are calculated over the same parameters of the individual measurements.

Category of measurement	Fields	Mean ± SD [%]	p value (*t*‐test)	Range [Min‐Max] [%]	Mean ± SD time [mm:ss]	Passed fields	Failed fields	Activated ICs [Mean ± SD]
**All irradiations**	23014 (100%)	−0.5 ± 0.9	*p* < 0.01	−19.4 – 17.3	03:24 ± 01:57	20909 (91%)	2105 (9%)	21 ± 2
**Tolerances passed**	20909 (91%)	−0.5 ± 0.8	*p* < 0.01	−7.0 – 7.0	03:25 ± 01:56	20909 (100%)	0	19 ± 2
**Tolerances failed**	2105 (9%)	−1.0 ± 1.3	*p* < 0.01	−19.4 – 17.3	03:15 ± 02:05	0	2105 (100%)	20 ± 2
**Ga treatment room**	9370 (41%)	−0.4 ± 0.9	*p* < 0.01	−19.4 – 17.3	04:12 ± 02:16	8497 (91%)	873 (9%)	20 ± 2
**Hx treatment room**	13640 (59%)	−0.6 ± 0.8	*p* < 0.01	−18.4 – 17.1	02:52 ± 01:28	12488 (92%)	1152 (8%)	20 ± 2
**All ^12^C fields**	10872 (47.2%)	−0.5 ± 0.8	*p* < 0.01	−19.4 – 17.3	03:46 ± 02:08	9963 (92%)	909 (8%)	20 ± 2
**All ^1^H fields**	11894 (51.7%)	−0.5 ± 0.9	*p* < 0.01	−19.0 – 15.1	03:06 ± 01:43	10720 (90%)	1174 (10%)	20 ± 2
**All ^4^He fields**	248 (1.1%)	−0.1 ± 1.0	*p* = 0.38	−10.0 – 12.2	01:42 ± 00:52	225 (91%)	23 (9%)	20 ± 2
**^12^C Ga**	4722 (20.5%)	−0.5 ± 0.8	*p* < 0.01	−19.4 – 17.3	04:29 ± 02:29	4235 (90%)	487 (10%)	20 ± 2
**^12^C Hx**	6146 (26.7%)	−0.5 ± 0.8	*p* < 0.01	−15.9 – 17.1	03:13 ± 01:36	5724 (93%)	422 (7%)	21 ± 2
**^1^H Ga**	4635 (20.1%)	−0.3 ± 1.0	*p* < 0.01	−19.0 – 15.1	03:55 ± 01:59	4250 (92%)	385 (8%)	20 ± 2
**^1^H Hx**	7259 (31.5%)	−0.7 ± 0.8	*p* < 0.01	−18.4 – 9.5	02:36 ± 01:17	6470 (89%)	789 (11%)	21 ± 2
**^4^He Ga**	13 (0.1%)	−1.0 ± 0.9	*p* < 0.01	−7.5 – 3.9	01:55 ± 01:07	12 (92%)	1 (8%)	19 ± 2
**^4^He Hx**	235 (1%)	0.0 ± 0.9	*p* = 0.95	−10.0 – 12.2	01:41 ± 00:51	214 (91%)	21 (9%)	20 ± 2
**RayStation TPS**	5267 (23%)	−0.2 ± 0.9	*p* < 0.01	−17.0 – 17.0	02:55 ± 01:46	4752 (90%)	515 (10%)	20 ± 2
**Syngo TPS**	17750 (77%)	−0.6 ± 0.8	*p* < 0.01	−19.4 – 19.0	03:36 ± 02:02	16204 (91%)	1546 (9%)	20 ± 2
**RaShi**	5621 (24%)	−0.7 ± 1.0	*p* < 0.01	−19.4 – 17.3	03:59 ± 02:09	5108 (91%)	513 (9%)	19 ± 2
**No RaShi**	17393 (76%)	−0.4 ± 0.8	*p* < 0.01	−19.0 – 17.1	03:13 ± 01:51	15801 (91%)	1592 (9%)	20 ± 2
**Abdomen**	2749 (12%)	−0.2 ± 0.9	*p* < 0.01	−17.0 – 14.8	05:48 ± 02:53	2558 (93%)	191 (7%)	20 ± 2
**Extremities**	474 (2%)	−0.2 ± 0.9	*p* < 0.01	−11.4 – 14.0	04:46 ± 02:16	453 (96%)	21 (4%)	19 ± 2
**Neck**	405 (2%)	−0.7 ± 0.8	*p* < 0.01	−19.4 – 13.1	03:07 ± 01:26	356 (88%)	49 (12%)	20 ± 2
**Thorax**	1164 (5%)	−0.4 ± 0.8	*p* < 0.01	−13.3 – 12.1	04:08 ± 01:46	1074 (92%)	90 (8%)	21 ± 2
**Pancreas**	415 (2%)	−0.7 ± 0.8	*p* < 0.01	−16.1 – 9.8	04:02 ± 01:53	387 (93%)	28 (7%)	20 ± 2
**Prostate**	599 (3%)	−0.6 ± 0.8	*p* < 0.01	−11.3 – 7.4	03:09 ± 00:50	573 (96%)	26 (4%)	20 ± 2
**Head**	15732 (68%)	−0.6 ± 0.8	*p* < 0.01	−18.4 – 17.3	02:51 ± 01:20	14129 (90%)	1603 (10%)	20 ± 2
**Spine and neuroaxis**	1391 (6%)	−0.2 ± 0.8	*p* < 0.01	−19.0 – 11.4	03:45 ± 01:57	1269 (93%)	95 (7%)	20 ± 2

*Note*: The values of the individual measurements include only the activated ICs. The percentages in the second column in parentheses refer to the total number of measurements (23014), while those in columns 7 and 8 refer to the respective numbers in column 2.

Abbreviations: ^12^C, carbon ions; Ga, gantry treatment room; ^1^H, protons; ^4^He, helium ions; Hx, horizontal rooms; IC, ionization chamber; Max, maximum; Min, minimum; RaShi, range shifter; SD, standard deviation; TPS, treatment planning system.

**TABLE 3:** Summary of the analyzed measurements over the past 10 years of clinical operation. The percentages in the ninth column in parentheses refer to the corresponding numbers in column 4, 5, and 6, while all other percentages refer to the total number of measured fields of each year.

Year	Evaluated patients	Measured fields	Ion species(^12^C/*^1^* H /^4^He)	Ga	Hx	Use of RaShi	No RaShi	Repeated irradiations	Irradiation time [hh:mm:ss]
**2016**	726	2285	**^12^C**: 1070 ***^1^* H**: 1215	852 (37%)	1432 (63%)	596 (26%)	1689 (74%)	**Hx**: 64 (4.5%) **Ga**: 38 (4.5%) **^12^C**: 50 (4.7%) ***^1^* H**: 52 (4.3%)	**Hx**: 73:17:05 **Ga**: 62:48:55 **^12^C**: 71:04:55 ***^1^* H**: 65:03:30
**2017**	646	2098	**^12^C**: 993 ***^1^* H**: 1105	942 (45%)	1156 (55%)	539 (26%)	1559 (74%)	**Hx**: 51 (4.4%) **Ga**: 37 (4%) **^12^C**: 28 (3%) ***^1^* H**: 60 (5.4%)	**Hx**: 56:51:34 **Ga**: 65:55:36 **^12^C**: 63:21:21 ***^1^* H**: 59:25:49
**2018**	655	2488	**^12^C**: 1168 ***^1^* H**: 1320	1066 (43%)	1422 (57%)	637 (26%)	1851 (74%)	**Hx**: 30 (2.1%) **Ga**: 33 (3.1%) **^12^C**: 21 (1.8%) ***^1^* H**: 42 (3.1%)	**Hx**: 82:54:39 **Ga**: 74:51:54 **^12^C**: 77:38:59 ***^1^* H**: 80:07:47
**2019**	649	2024	**^12^C**: 1038 ***^1^* H**: 986	684 (34%)	1340 (66%)	460 (23%)	1564 (77%)	**Hx**: 39 (2.9%) **Ga**: 16 (2.3%) **^12^C**: 30 (2.9%) ***^1^* H**: 25 (2.5%)	**Hx**: 71:17:03 **Ga**: 51:46:38 **^12^C**: 71:11:45 ***^1^* H**: 51:51:56
**2020**	643	2504	**^12^C**: 1154 ***^1^* H**: 1350	947 (38%)	1557 (62%)	480 (19%)	2024 (81%)	**Hx**: 56 (3.6%) **Ga**: 21 (2.2%) **^12^C**: 39 (3.4%) ***^1^* H**: 38 (2.8%)	**Hx**: 80:56:16 **Ga**: 73:15:50 **^12^C**: 80:46:35 ***^1^* H**: 73:25:31
**2021**	610	2368	**^12^C**: 1157 ***^1^* H**: 1211	1109 (47%)	1259 (53%)	635 (27%)	1733 (73%)	**Hx**: 20 (1.6%) **Ga**: 22 (2.0%) **^12^C**: 18 (1.6%) ***^1^* H**: 24 (2.0%)	**Hx**: 62:20:02 **Ga**: 81:31:59 **^12^C**: 74:46:12 ***^1^* H**: 69:05:49
**2022**	624	2677	**^12^C**: 1282 ***^1^* H**: 1395	1192 (45%)	1485 (55%)	719 (27%)	1958 (73%)	**Hx**: 22 (1.5%) **Ga**: 21 (1.8%) **^12^C**: 18 (1.4%) ***^1^* H**: 25 (1.8%)	**Hx**: 63:50:37 **Ga**: 83:52:33 **^12^C**: 77:40:19 ***^1^* H**: 70:02:51
**2023**	652	2379	**^12^C**: 1104 ***^1^* H**: 1265 **^4^He**: 10	1067 (45%)	1312 (55%)	608 (26%)	1771 (74%)	**Hx**: 37 (2.8%) **Ga**: 39 (3.7%) **^12^C**: 47 (4.3%) ***^1^* H**: 29 (2.3%) **^4^He**: 0 (0%)	**Hx**: 64:35:36 **Ga**: 69:58:24 **^12^C**: 71:54:37 ***^1^* H**: 62:25:53 **^4^He**: 0:13:30
**2024**	620	2477	**^12^C**: 1110 ***^1^* H**: 1262 **^4^He**: 105	1013 (41%)	1464 (59%)	601 (24%)	1876 (76%)	**Hx**: 36 (2.5%) **Ga**: 34 (3.4%) **^12^C**: 39 (3.5%) ***^1^* H**: 27 (2.1%) **^4^He**: 4 (3.8%)	**Hx**: 59:29:21 **Ga**: 60:25:59 **^12^C**: 63:11:56 ***^1^* H**: 53:34:50 **^4^He**: 3:08:34
**2025 (01‐06)**	347	1538	**^12^C**: 629 ***^1^* H**: 776 **^4^He**: 133	581 (37%)	957 (63%)	392 (25%)	1146 (75%)	**Hx**: 6 (0.6%) **Ga**: 3 (0.5%) **^12^C**: 4 (0.6%) ***^1^* H**: 5 (0.6%) **^4^He**: 0 (0%)	**Hx**: 37:19:00 **Ga**: 35:05:27 **^12^C**: 34:40:04 ***^1^* H**: 34:06:29 **^4^He**: 3:37:54
**Total**	6348	23014	**^12^C**: 10805 ***^1^* H**: 11961 **^4^He**: 248	9453 (41%)	13557 (59%)	5667 (25%)	17347 (75%)	**Hx**: 361 (2.7%) **Ga**: 264 (2.8%) **^12^C**: 294 (2.7%) ***^1^* H**: 327 (2.7%) **^4^He**: 4 (1.6%)	**Hx**: 652:51:13 **Ga**: 659:33:15 **^12^C**: 686:16:43 ***^1^* H**: 619:10:25 **^4^He**: 06:59:58

Note: Note that measured fields don't include the number of repeated measurements, and the number of evaluated patients can differ from the number of treated patients in that time period.

Abbreviations: ^12^C, carbon ions; ^1^H, protons; ^4^He, helium ions; Ga, gantry treatment room; Hx, horizontal treatment room; RaShi, range shifter; SD, standard deviation.

The passing rate of the dose verification measurements according to the criteria in Table 1 was 91 % (99.28 ± 0.40 % of all activated ICs within ±7 % (Table S1)). The highest passing rates were observed for prostate and extremities with 96 % followed by abdomen, pancreas, and spine while the lowest was for the neck (88%) and head (90%) groups. In all categories presented in Table 2, at least 19 ± 2 ICs of the 24‐array were activated and included in the dosimetric analysis. Further, Table S1 displays the number of individual measurement points (activated and deactivated) with deviations below certain thresholds. On average, 2.72 % of the activated ICs were outside the ±5 % thresholds and only 0.72 % outside ±7 % tolerance limits for the maximum and minimum doses, respectively. The number of activated ICs was stable over time and ranged between 82% and 85 % of a total of 551350 measured points over the reported period. Furthermore, 13 ± 1.0 % and 15 ± 0.9 % of the deactivated ICs were within the thresholds ± 5 % and ± 7 %, respectively.

**FIGURE 4:**
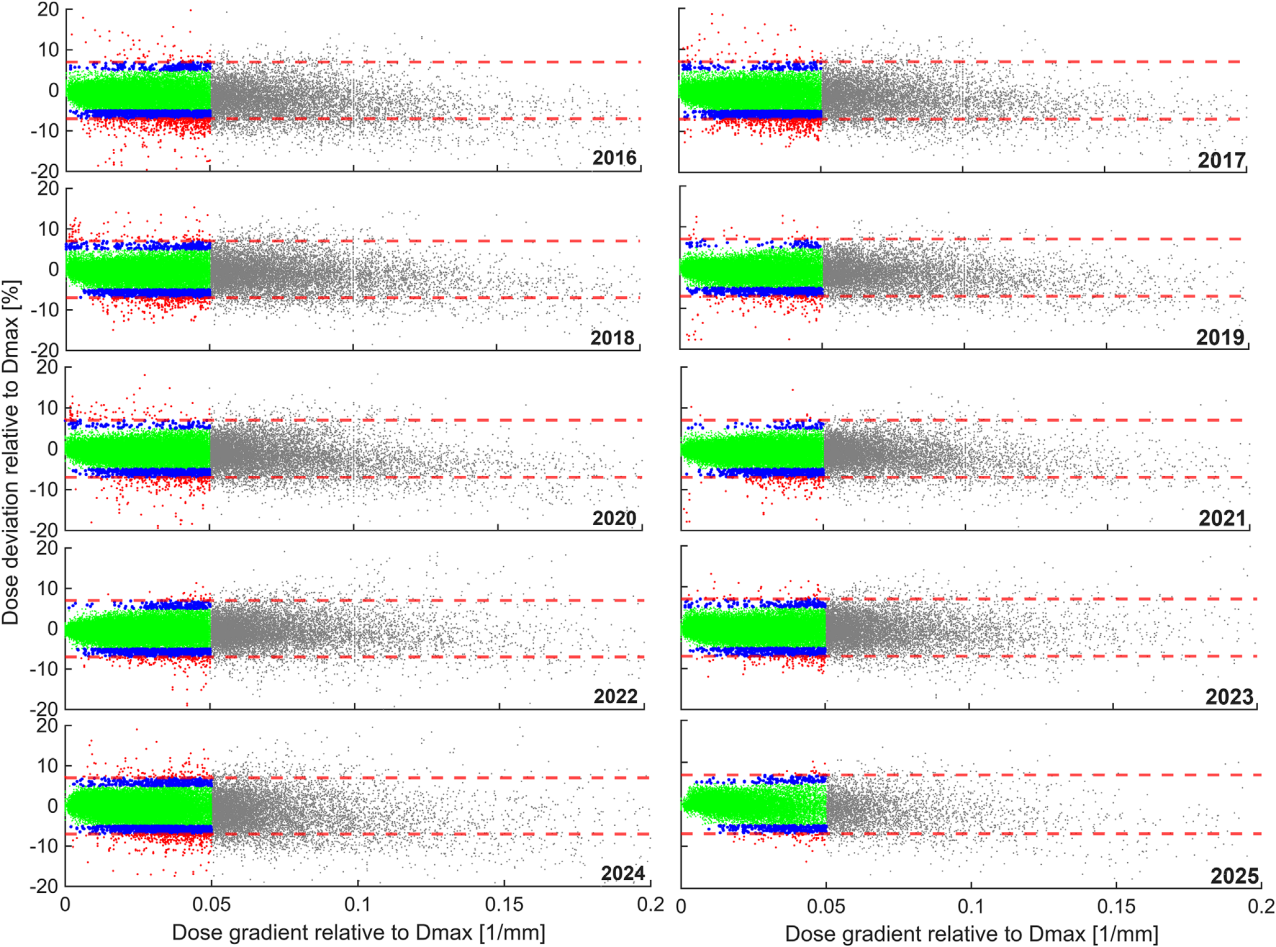
Distribution of the dose deviations of the 24 individual ICs obtained from all measurements per year as a function of the dose gradient at the IC position. Green points present activated ICs, blue activated ICs between ±(5–7)%, red activated ICs beyond ±7 % and gray deactivated ICs. The tolerance levels are presented by dashed lines (±7 % for the minimum and maximum deviation). *D*_max_  maximum dose in each field; IC, ionization chamber.

Figure 4 visualizes the distribution of the dose deviations of the individual deactivated and activated 24 ICs obtained from all measurements per year as a function of the dose gradient at the IC position.

### 3.2 Irradiation time

Table 2 also displays the recorded mean irradiation time per field for different categories. Preparation and analysis time are not included. In total, more than 1312 h (i.e., 164 working days) have been spent on dose verification measurements at HIT over the last 10 years. Although ^12^C verifications required on average 18 % more beam time than protons, as shown in the same table, more beam time per fields was used in the gantry room (04:12  ±  02:16 [mm:ss]) compared to Hx (02:52  ±  01:28 [mm:ss]). Table 4 presents the mean irradiation time per field over the past 10 years. Less beam time per field has been used from 2023 on, while the previous years showed stable mean ± SD irradiation times. Comparing the different indications (Table 2), the abdomen group showed the highest irradiation time with 05:48 ± 02:53 [mm:ss] while the lowest was found for the head group with 02:51 ± 01:20 [mm:ss].

**TABLE 4:** Summary of mean ± SD of the deviations, irradiation time per measurement and number of activated ICs over the last 10 years. Data include all rooms, ion species and indications, independently of the use of the RaShi.

Year	Mean ± SD dose deviation [%]	p value (*t*‐test)	Mean ± SD irradiation time [mm:ss]	Mean ± SD of activated ICs
**2016**	−0.9 ± 1.5	*p* < 0.01	03:34 ± 01:56	20 ± 2
**2017**	−0.8 ± 1.6	*p* < 0.01	03:32 ± 02:12	20 ± 2
**2018**	−0.5 ± 0.9	*p* < 0.01	03:33 ± 01:55	20 ± 2
**2019**	−0.6 ± 0.8	*p* < 0.01	03:39 ± 02:09	20 ± 2
**2020**	−0.6 ± 0.8	*p* < 0.01	03:42 ± 02:15	20 ± 2
**2021**	−0.6 ± 0.7	*p* < 0.01	03:39 ± 01:52	20 ± 2
**2022**	−0.5 ± 0.8	*p* < 0.01	03:19 ± 01:53	20 ± 2
**2023**	−0.3 ± 0.8	*p* < 0.01	03:09 ± 01:52	21 ± 2
**2024**	−0.2 ± 1.0	*p* < 0.01	02:54 ± 01:43	21 ± 2
**2025 (01‐06)**	−0.1 ± 0.9	*p* < 0.01	02:50 ± 01:51	20 ± 3
**Total**	−0.5 ± 0.9	*p* < 0.01	03:24 ± 01:59	20 ± 1

Abbreviations: *D*_max_, maximum physical dose in the irradiation field; IC, ionization chamber; RaShi, range shifter; SD, standard deviation; TPS, treatment planning system.

### 3.3 Dosimetric results

**FIGURE 5:**
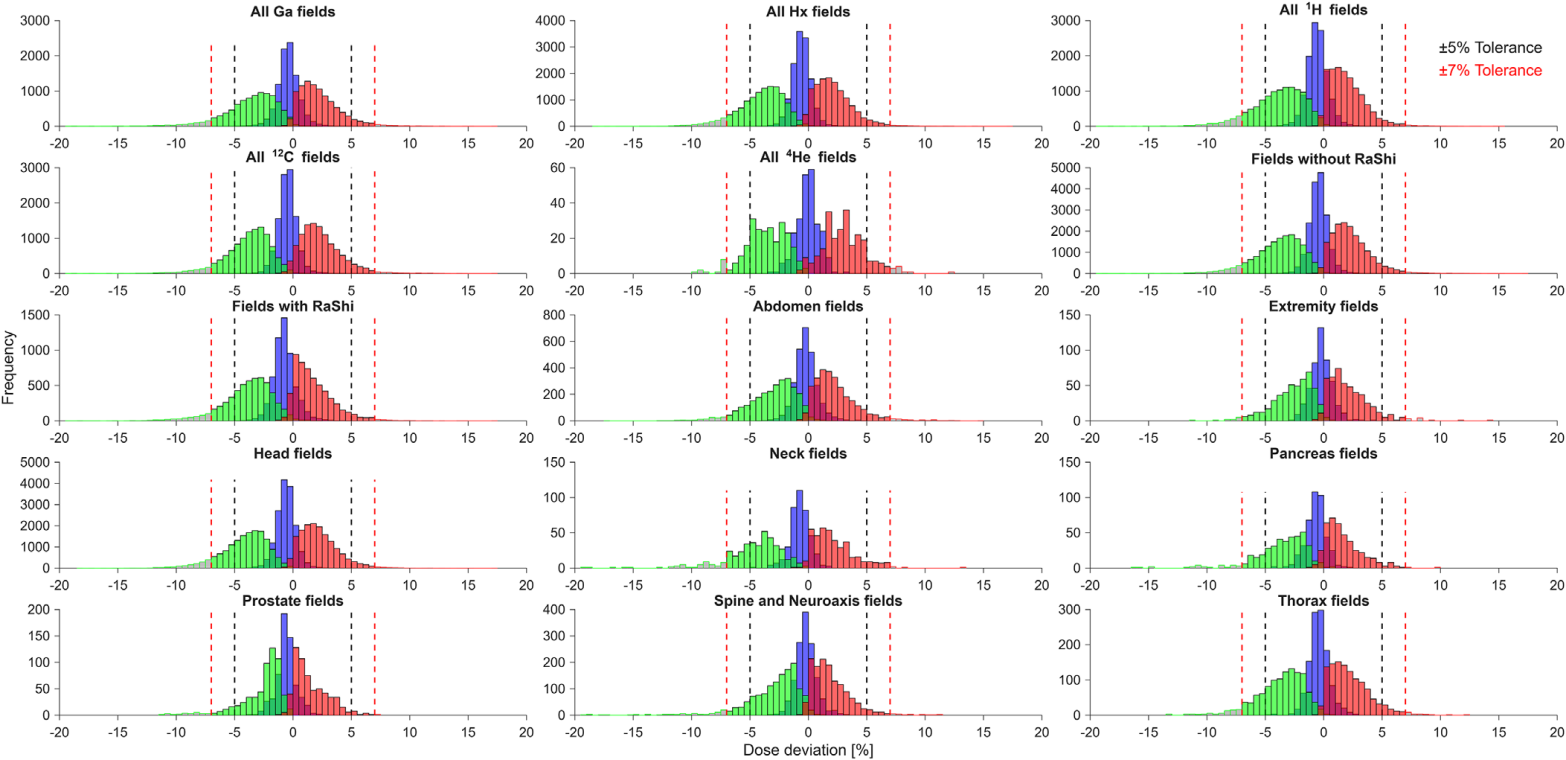
Frequency histograms showing the mean (blue bars), maximum (red bars) and minimum (green bars) dose deviations for different groups of measurements. Bars outside the tolerance range are shown in gray with colored edges corresponding to their data type (blue for mean, red for maximum, green for minimum). Overlapping values are highlighted with special colors: red + blue + green = magenta, red + green (without blue) = orange, red + blue = purple, and blue + green = cyan. The tolerance levels are presented by dashed lines (black ± 5 % for the mean, red ± 7 % for the minimum and maximum deviation). ^12^C, carbon ions; Ga, gantry treatment room; ^1^H, Protons; ^4^He, helium ions; Hx, horizontal rooms; RaShi, range shifter.

To assess the dosimetric quality of our dose verification strategy, the measurement data were analyzed separately according to treatment room, ion species, TPS, use of RaShi, indication, depth, target volume, and repeated measurements as well as combinations of these parameters (Table 2). For all investigated categories, the total mean ± SD dose deviations were within ±1 %. Additionally, Figure 5 displays the frequency distributions of the measured deviations (mean, maximum, and minimum) for the different categories. Only the mean dose deviations for ^4^He (Ga and Hx) were found to be normally distributed (Shapiro–Wilk test, P > 0.01), however, the distributions were highly symmetric for all categories. The deviations of the total mean deviation from zero were found to be statistically significant (*t*‐test, *p* < 0.01) for all investigated categories, except for “all ^4^He fields” and “^4^He Hx” (Table 2). Also, the median of the maximum and minimum deviations differed significantly from zero (Wilcoxon signed‐rank test, *p* < 0.01).

#### 3.3.1 Treatment room

The total mean ± SD dose deviations [range of *D*_min_ and *D*_max_] were −0.4 ± 0.9 % [−19.4 – 17.3] for the Ga room and −0.6 ± 0.8 % [−18.4 – 17.1] for the Hx rooms (Table 2). Moreover, while carbon ions show similar mean ± SD dose deviations in Hx and at the Ga, protons show a reduced mean ± SD dose deviations at the Ga compared to Hx with −0.3 ± 1.0 % and −0.7 ± 0.8 %, respectively. Furthermore, the treatment rooms (Hx and Gantry) are statistically significant (Welch *t*‐test, *p* < 0.01).

#### 3.3.2 Ion species

**FIGURE 6:**
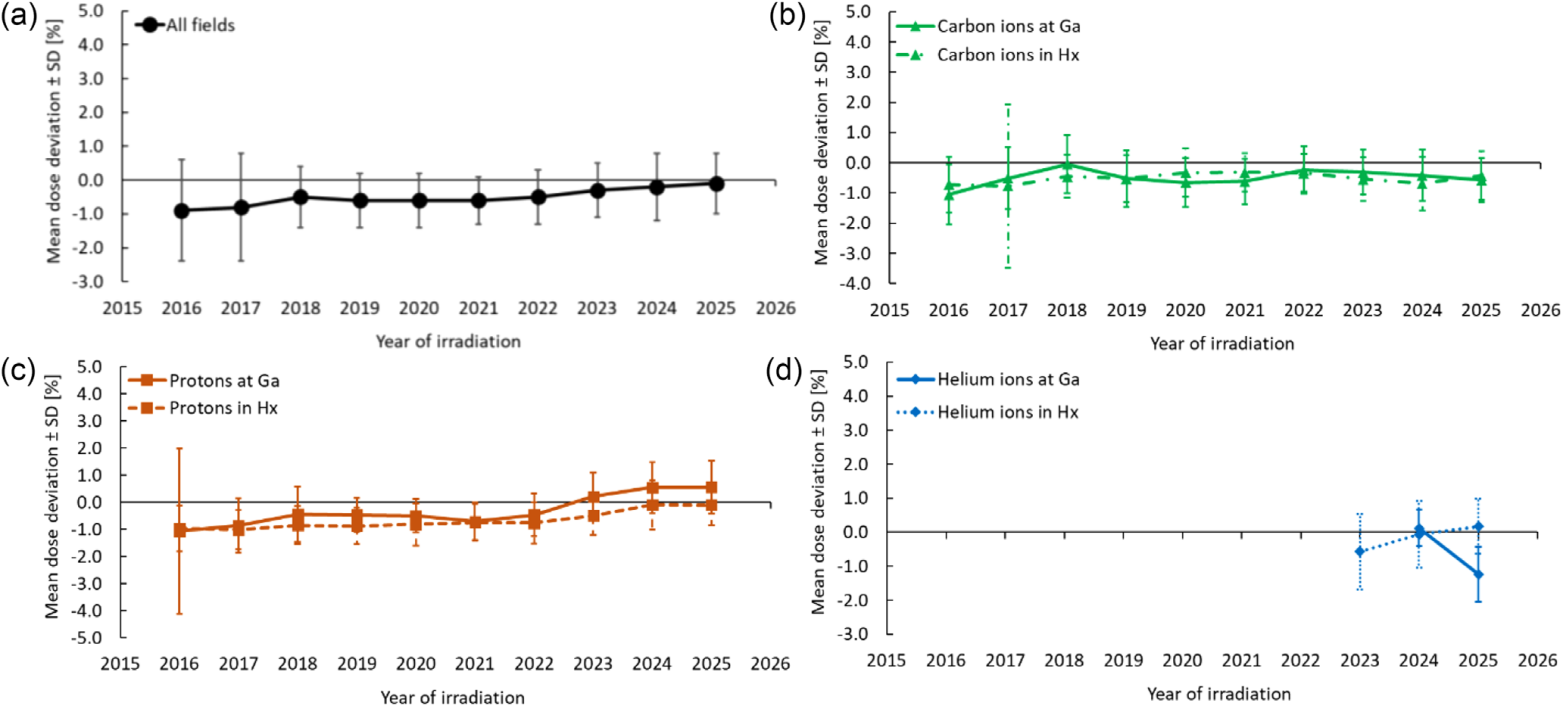
Total mean dose deviations ± SD over all individual measurements at HIT during the past 10 years. In 2023, the Syngo TPS was replaced by RayStation. Ga, gantry treatment room; Hx, horizontal treatment room; SD, standard deviation.

The total mean ± SD dose deviations [range of *D*_min_ and *D*_max_] were −0.5 ± 0.9 % [−19%–15.1 %] for ^1^H, −0.5 ± 0.8 % [−19.4%–17.3 %] for ^12^C, and −0.1 ± 1.0 % [−10%–12.2 %] for ^4^He (Table 2). Figure 6 shows the total mean ± SD of all individual mean dose deviations recorded in the past 10 years. A tendency for improvement over time was found for protons in the Hx and Ga treatment rooms. Total mean deviations ranged between −1 % and 0 %, independently of the used treatment room and ion species.

Furthermore, the differences between the ion species (protons vs. helium vs. carbon ions) were statistically significant (Anova and Tukey HSD, *p* < 0.01). In addition, the differences between ^12^C Ga, ^12^C Hx, ^1^H Ga, ^1^H Hx, ^4^He Ga, and ^4^He Hx (Table 2) were found to be statistically significant (ANOVA and Tukey HSD, *p* < 0.01), except for ^12^C Ga vs. ^12^C Hx, ^12^C Ga vs. ^4^He Ga, ^12^C Hx vs. ^4^He Ga, and ^1^H Hx vs. ^4^He Ga.

#### 3.3.3 Range shifter

Table 2 shows that the total mean ± SD dose deviations [range of *D*_min_ and *D*_max_] with RaShi were significantly larger (−0.7 ± 1 % [−19.4–17.3]) than without RaShi (−0.4 ± 0.8% [−19–17.1]) (Welch *t*‐test, *p* < 0.01). However, fields with or without RaShi show the same passing rate of 91%.

#### 3.3.4 TPS

Table 2 shows that 77 % of the analyzed irradiation fields were planned using the Syngo TPS. The total mean dose deviations were smaller for the calculation with the RayStation TPS (−0.2 ± 0.9%) than for the Syngo TPS (−0.6 ± 0.8%) (Welch *t*‐test, *p* < 0.01). Further, Figure 6 shows that this difference in mean dose deviations between both TPSs is coming from protons since the use of the RayStation TPS from 2023 on.

#### 3.3.5 Indication

**FIGURE 7:**
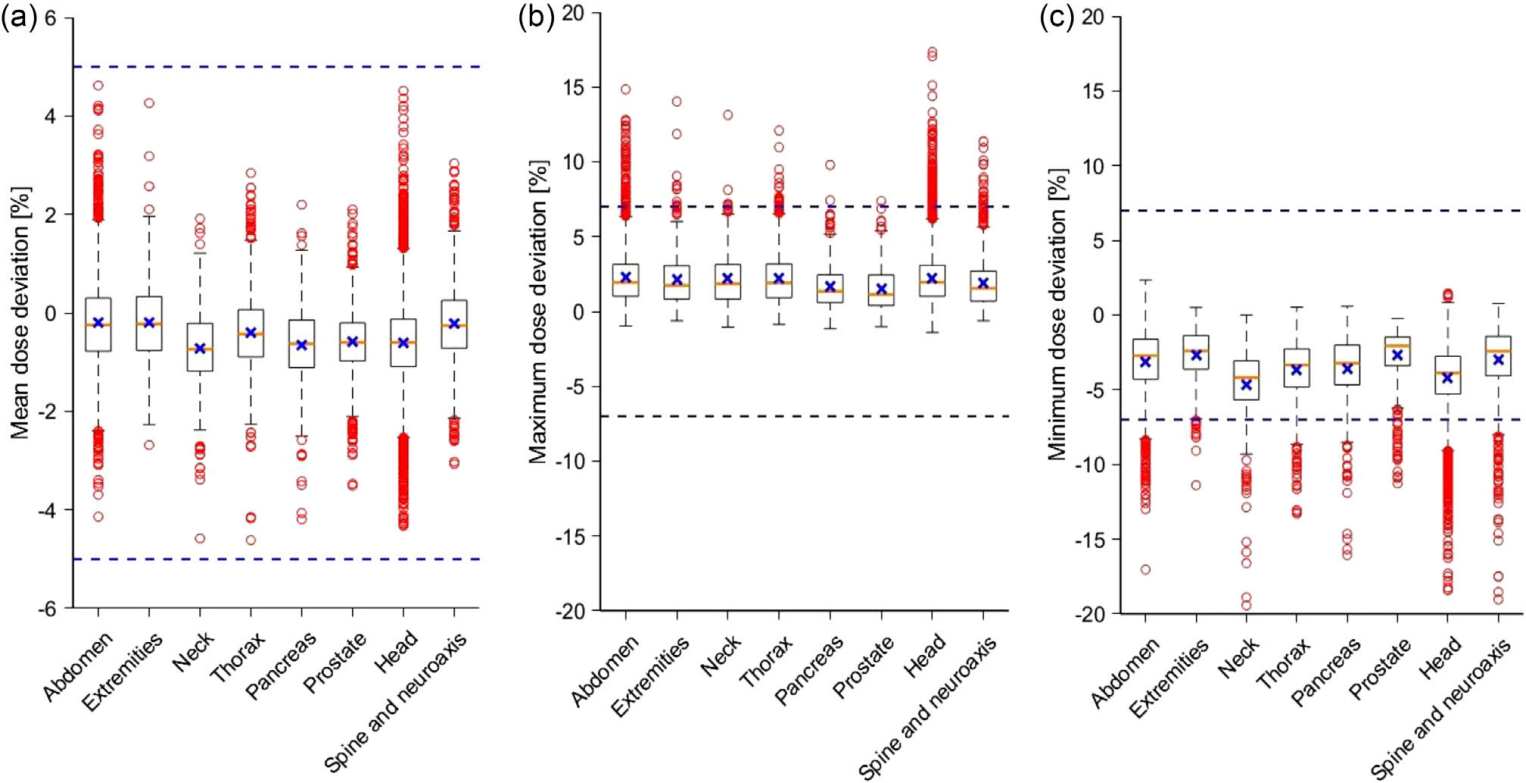
Comparison of the distributions of mean (a), maximum (b) and minimum (c) dose deviations, obtained in the measurements of the different indications (yellow line = median, x = mean, box = interquartile range, whiskers = 1.5 x interquartile range, circles = outliers). ± 5% and ±7 % tolerances are indicated by blue dashed lines.

Figure 7 shows a comparison of the distributions of mean, maximum, and minimum dose deviations for the different indications measured in the last 10 years. Numerical values are depicted in Table 2 and show total mean deviations between −0.2 % and −0.7 %.

In contrast to the mean deviations, most outliers for the minimum and maximum deviations were found outside the ± 7% tolerance limits. However, all median deviations are within ± 5%. Pairs of mean dose deviations differed significantly between all indications (Anova and Tukey HSD, *p* < 0.01), except abdomen vs. extremities, abdomen vs. spine, extremities vs. spine, head vs. neck, head vs. pancreas, head vs. prostate, neck vs. pancreas, neck vs. prostate, and pancreas vs. prostate (Figure 7).

#### 3.3.6 Depth

**FIGURE 8:**
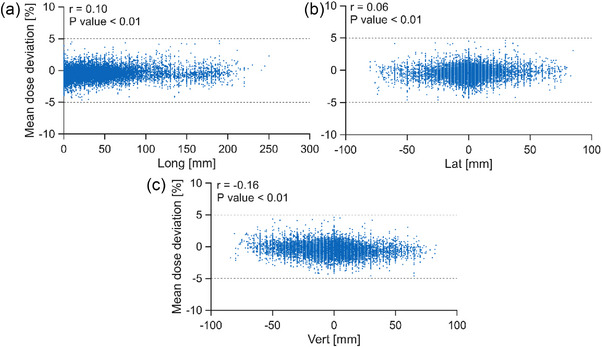
Dependence of the mean dose deviations obtained in the individual measurements as a function of the IC stack position in longitudinal (a), lateral (b), and vertical (c) direction. The origin refers to the center of the WP entrance window positioned at the isocenter. The longitudinal axis represents the depth in water. The Pearson correlation coefficient (*r*) and the respective p values are also given. The dashed lines illustrate the ±5 % tolerances.

Figure 8 displays the mean deviations observed in the dose verification measurements as a function of the IC stack position in the WP in longitudinal, lateral, and vertical direction. Using the Pearson correlation, a significant, but very weak correlation between the mean dose deviation and array position was found.

#### 3.3.7 Target volume

**FIGURE 9:**
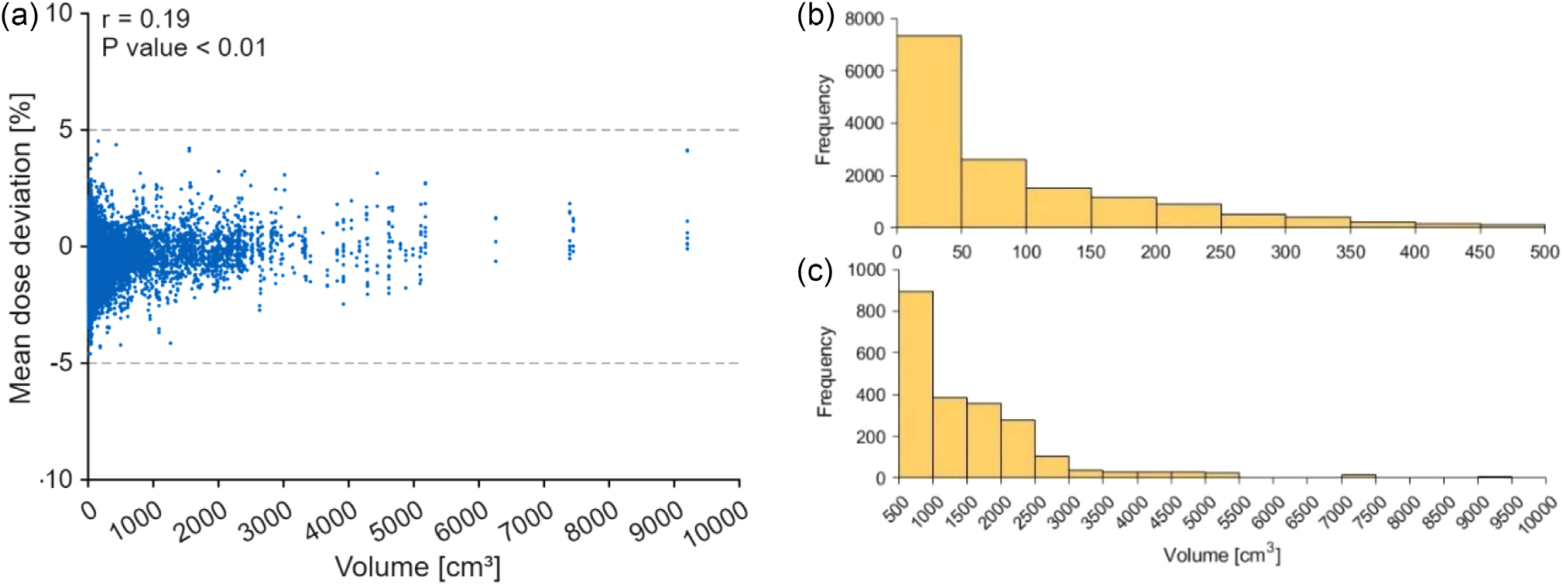
(a) Distribution of the mean dose deviations obtained in the individual measurements as a function of target volume. The Pearson correlation coefficient (*r*) and p values are also given. The dashed lines illustrate the ±5 % tolerances for the mean dose deviation. In addition, frequency histograms of smaller (b) and larger (c) target volumes are displayed.

Figure 9a presents the distribution of the mean dose deviations obtained in the individual measurements as a function of target volume. The Pearson correlation coefficient indicates a significant, but very weak correlation between the mean deviations and the size of the target volume. Figure 9b,c illustrate that the frequency of volumes decreases with volume.

#### 3.3.8 Original versus repeated verification

Only around 3 % of the total dose verification measurements in the last 10 years were repeated because of assumed human operating errors and technical issues. Moreover, a measurement was repeated if (i) there were unexpectedly large mean dose deviations compared to previous verifications in the same category, (ii) maximum and minimum dose deviations exceed ± 7%–10 %, e.g., if highly modulated fields were applied.

**FIGURE 10:**
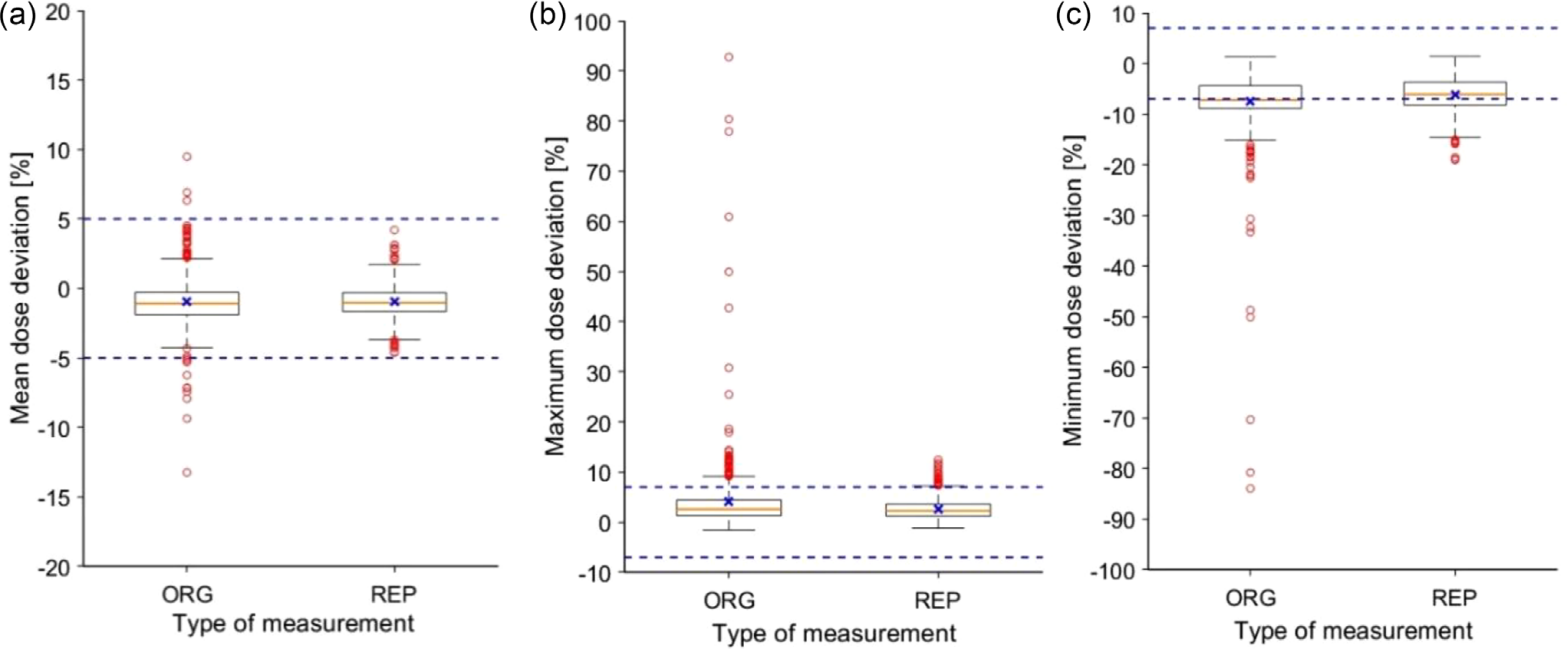
Comparisons of the distributions of mean (a), maximum (b), and minimum (c) dose deviations, obtained in original and repeated measurements over the last 10 years (yellow line = median, x = mean, box = interquartile range, whiskers = 1.5 x interquartile range, and circles = outliers). ± 5% and ±7 % tolerance limits are indicated by blue dashed lines. ORG, original measurement; REP, repeated measurement.

Figure 10 shows a comparison of the distributions of mean, maximum, and minimum dose deviations obtained in original and repeated measurements over the last 10 years. A reduced variation of the mean, minimum, and maximum dose was noticed when repeating the measurements. However, there are still minimum and maximum outliers out of the ±7 % tolerances.

Note that the number of repeated measurements may be larger than that one of original measurements since a treatment field may be measured several times during the repetition. Table S2 summarizes the results of the original and repeated measurements. The total mean dose deviations of the original and repeated results from zero were found to be statistically significant (*t*‐test, *p* < 0.01), while there was no statistically significant difference between the original and repeated measurements (*t*‐test, *p* > 0.05).

## 4 DISCUSSION

PSQA is crucial when using scanned pencil beams in particle therapy.^13^ The dose delivered by a treatment field depends on the number of ions, accurate monitor calibration, spill shape (i.e., beam position and width drifts during one energy extraction), beam position and width deviations from the nominal values, which are used in the TPS. Such deviations can significantly alter the dose distribution, affecting dose homogeneity in the target, target conformity, and dose to OAR. For this purpose, 2D arrays, flat panel detectors for fluence measurements and subsequent reconstruction of 3D absorbed dose distribution^3^, ^12^, ^16^, ^32^, ^33^ or a detector array‐equipped WP for simultaneous point dose measurements^13^, ^15^ can be used.

At HIT, the planned dose in the TPS is checked for a sample of positions either by a measurement in the WP or an IDC prior to the first treatment fraction to ensure accurate beam delivery throughout the treatment field. Advantages of such PSQA measurements include the use of identical patient treatment plans as those delivered during treatment, with nearly the same beam optics and machine behavior. However, such absolute dosimetric verifications demand high personnel resources and are time‐consuming in terms of beam and room time, making them overall very cost‐intensive.

Further, the dosimetric quality and accuracy of PSQA measurements in WPs are limited by (i) the uncertainty of the effective point of measurement of the IC, (ii) possible positioning errors of the IC PMMA mounting of the IC as well as the WP, and (iii) the extended sensitive volume of the IC. Although (i) and (ii) will lead to changes of the measured doses in steep dose gradients, (iii) will lead to an averaging over the sensitive volume and altogether they may lead to wrong determination of the dose at the IC position.^12^, ^13^ Moreover, the current WP design only allows measuring in horizontal beams. Additionally, in some cases PSQA measurements are generally not feasible, e.g. in the context of online adaptive particle therapy (APT), where treatment plans are modified based on CT or magnetic resonance imaging (MRI) scans, while the patient lies on the treatment couch.^34^

A general limitation of this type of PSQA is also, that only the dose in a homogeneous medium can be measured and any patient related effects are excluded. This includes specifically a dosimetric effect of large tissue inhomogeneities, which is a typical challenge for dose algorithms. Moreover, the measurements are point‐doses integrated over all energies and scan spots, which do not allow any specific conclusion about potential delivery errors related to beam characteristics. Last not least, measurements are usually only performed once and delivery accuracy is assumed to remain constant over the full course of treatment. Therefore, alternative PSQA approaches are currently of great interest in particle therapy, such as IDC^17^, ^18^, ^19^, ^20^, ^35^, ^36^, ^37^, ^38^ and machine log file‐based^21^, ^22^, ^23^, ^24^, ^39^ PSQA. Although independent dose calculations and log‐file analysis are promising complementary approaches for PSQA, they are not included in this study. Implementation of these methods is ongoing at HIT and should in the future help reduce the need for routine phantom measurements. In the last 10 years, these methods were investigated as a potential substitute for the phantom measurements, enabling validation of control parameters independently of the treatment planning system. However, there are no guidelines recommending specific PSQA methods to particle therapy.

The European Particle Therapy Network (EPTN), which held a workshop on this PSQA topic in 2024, is currently working on a guideline for PSQA in particle therapy. In contrast, various studies, reports and guidelines investigated and summarized PSQA procedures, its failure modes, errors and risk analysis in photon therapy.^40^, ^41^, ^42^, ^43^, ^44^ Currently, application of PSQA for ion beam therapy is based on these studies and guidelines due to the lack of ion‐specific studies. To fill this gap for ion beam therapy, we analyzed the WP measurements performed over the past 10 years for protons, carbon, and helium ions.

### 4.1 Measurement characteristics

Based on the evaluated measurement characteristics (Table 2), the overall passing rate of 91 % remained high and clinically acceptable across all indications. Here, the observed outliers with deviation of more than ± 7% for the maximum and/or minimum dose deviations are mostly connected to the three uncertainties (i)–(iii) mentioned above, which are relevant especially in regions with steep dose gradients. For instance, highly modulated treatment fields are assumed to show more outliers. In contrast, for the prostate and extremities groups, less modulated fields due to the location of the adjacent OARs are applied, thus showing the highest passing rates with 96 %. Furthermore, the total passing rate was found to depend on the applied gradient threshold. It increased up to 95 % when excluding more ICs by using a stricter threshold of 4 % of *D*_max_ for deactivating ICs with high dose gradients instead of the 5 % applied threshold.

Additionally, the mean dose deviations were found to be statistically significant (*t*‐test, *p* < 0.01) for most groups. Exceptions were observed for “all ^4^He fields” and “^4^He Hx”, where the mean deviations were close to zero and the variability across measurements was relatively large, resulting in nonsignificant p values. In contrast, small groups such as “^4^He Ga” showed significant deviations because the observed differences were larger relative to their measurement variability, leading to *p* < 0.01 despite the limited sample size.

An interesting finding (Table 2 and Figure 4) was the stable and highly reproducible number of activated ICs per measurement (19 ± 2 ICs). On average, only 0.72 % of these activated ICs were out of the ±7 % tolerance limits for the maximum and minimum doses, indicating that the machine QA underlying the PSQA was consistently within the established tolerance levels throughout the analyzed period, ensuring reliable and reproducible verification measurements. Additionally, it should be noted that various indications and optimization techniques were used in the analyzed period, and the verification measurements were robust for all different types of plans.

### 4.2 Irradiation time

As shown in Table 2 and Table 3, current PSQA at HIT is time consuming. Besides the specified times, the TPS preparation time (ca. 15 min per verification), WP setup time (ca. 30 min per irradiation session), preirradiation time (ca. 5 min per irradiation session) and analysis time (10 min per verification) has to be considered. If these additional times are considered, the total time for PSQA in these 10 years is approximately 4308 h (2996 h for preparation and analysis, 1312 h for measurements). Thus, there is a need to improve the PSQA strategy at HIT to make it more efficient. Potential irradiation technique which may become extremely time consuming with the present PSQA measurements are e.g. particle arc therapy with step‐and‐shoot delivery using many gantry angles^45^ and APT on daily basis,^46^ where the treatment plans are modified, applied on the fly and verified afterwards. In addition, the reduction in verification time since 2023 is attributed to workflow and organizational optimizations in the beam application process, mainly related to a reduced need for beam interlock resets and improved delivery sequence. Furthermore, since more larger target volumes were treated with carbon ions than with protons, 18 % more beam time was used for carbon‐ion PSQA measurements. Additionally, more irradiation time is required for gantry plans, as most body treatments are delivered with the gantry and involve the largest treatment volumes.

### 4.3 Dosimetric results

Our dosimetric results showed a very good agreement in terms of mean deviation between measured and planned doses when excluding IC positions with gradients of more than 5 % of *D*_max_. Mean dose deviations were within 1 %, independent of the treatment room, ion species, RaShi, TPS, and indication. The observed mean deviations within 1 % correspond to dose differences of only a few milligray in typical clinical fractions (for example, 0.3 % of 2 Gy = 6 mGy), which is far below any clinically relevant threshold and confirms the high dosimetric stability of the delivery system. This is attributed to our high‐level periodic machine QA, which assures the settings of the delivery system for the whole spectrum of applications. The only difference is the passing rate, which is connected to the complexity and degree of dose modulation in the treatment plans, and to the resulting impact of the specific uncertainties (i)–(iii) when using the WP.

Comparable large‐scale PSQA investigations at other ion beam therapy centers have reported comparable levels of dosimetric accuracy. For example, Lima et al.^47^ conducted Monte Carlo‐based verification of proton treatment plans at CNAO. They found mean deviations of less than 1% when comparing simulated and measured doses in a water phantom setup. Similarly, G Magro et al.^48^ demonstrated an average deviation of less than 1% and excellent reproducibility for scanned proton beams, thereby validating the accuracy of treatment planning through both Monte Carlo and experimental measurements. The present 10‐year analysis shows that mean dose deviations remained within ±1% across more than 23000 proton, carbon, and helium ion fields. This confirms that the long‐term performance of PSQA at HIT is consistent with, and extends, the previously published benchmarks for scanned‐beam ion therapy.

Furthermore, a slight reduction in mean dose deviation was observed for protons in both the Hx and Ga treatment rooms after 2023 (Figure 6), coinciding with the clinical transition from the Syngo to the RayStation TPS. The implementation of the Monte Carlo calculation in RayStation improved proton dose calculations, particularly in situations involving beam modifiers such as range shifters or large air gaps, reducing systematic deviations from about –3% to less than –1%. For carbon ions, both TPSs produced comparable results, confirming consistent dose calculation accuracy across systems. Overall, the transition to RayStation yielded a modest but systematic improvement in proton dose agreement while maintaining stable dosimetric performance for all ion species within ±1%.

In addition, the results showed a very weak correlation between the mean dose deviation and the array position in all three directions as well as with the size of the target volume. Although the correlation was statistically significant, the related change in dose deviations remained clinically negligible. This indicates that the results of the verification measurements are essentially independent of target depth, volume as well as delivered energy and particle numbers.

In general, mean deviations between several analyzed categories differed significantly, although the difference itself was small and clinically negligible. These findings can be attributed to the large sample sizes allowing detection of even very small but insignificant differences. Although there may be a subtle reason for these differences, none of the categories performed particularly bad with respect to mean dose deviation. Failure of plan verification was therefore mainly induced by large deviations at single measurement positions, potentially induced by uncertainties in IC positioning as well as dose calculation and delivery in steep dose gradients. When deviations beyond ±7% occurred, the treatment plan was reviewed to identify the cause. If only a few chambers were affected, and if the deviations could be explained by steep dose gradients or measurement geometry, the results were accepted without modification to the plan.

In contrast, if deviations beyond ±7% were observed in several chambers and these could not be explained by physical or geometrical factors, the plan was re‐evaluated and adapted, if necessary, before treatment. This approach ensures that the deviations remain within clinically acceptable limits, and that PSQA findings are consistently incorporated into the decision‐making process. Furthermore, it is worth mentioning that in only a very few cases (estimated at around 10), the treatment plan was not delivered at all when the limit tolerances of ±7 % were exceeded.

As an additional reason of failure, it has to be noted that the uncertainty of the dosimetric system used in this study is not negligible. Measurements involve very low signal levels and a multichannel readout, which does not match the precision of reference dosimetry systems (e.g., a single channel electrometer together with a farmer ionization chamber). Furthermore, the process of background subtraction, especially in earlier measurements, has introduced some variability in the IC signals and systematic shifts to negative values, possibly contributing to the observed slightly negative mean deviations. These factors may explain some of the residual variation and outliers.

Finally, repeating measurements that initially failed showed that although there was an improvement in the mean dose deviation, there were still maximum and minimum dose deviations exceeding the ±7 % tolerances limits (Table S2). This indicates that the conditions that cause these large dose deviations are stable and lead to largely reproducible verification results. To better understand and identify the conditions under which dose deviations of more than ±7 % can occur (Figure 4 and Table S1), further investigations have to be performed using complementary high‐resolution 2D detector measurements. These measurements allow comparison of lateral dose profiles at relevant depths with TPS calculations, helping to distinguish whether deviations originate from TPS modeling, beam delivery, or phantom setup. Furthermore, future work could include cross‐validation between single channel and multichannel dosimeters, using the same chamber position, to better quantify and possibly correct for the measurement‐related uncertainties.

### 4.4 Consequences in clinical practice

#### 4.4.1 Necessity of measurements

An important question is whether dosimetric patient plan verifications in ion beam therapy prior to the first irradiation should be continued and if so, to what extent. The methods to verify the dose distribution are mainly described in the literature for photon radiotherapy.^49^ Methods of this verification are either dosimetric verification measurements, preferably performed with high‐resolution detector arrays, or independent dose recalculations of the patient‐specific treatment plan. To date, no recommendation for PSQA in particle therapy exists, but would be of great interest. The ESTRO particle therapy network, EPTN, has recently held a workshop on this topic and established a committee working on such a guideline.^50^

Based on the data analyzed in this work (Table 2), the high passing rates of the dosimetric PSQA (above 90%) in the past 10 years suggests, that reducing the number of time‐consuming measurements might be possible, if the current high‐level routine machine QA program is continued. This ensures that deviations of the beam delivery parameters are within institutional tolerance limits and may significantly reduce the logistic effort while still assuring safe dose delivery.

The value of using IDC methods instead of PSQA measurements as a part of the entire PSQA has been investigated. It has been concluded that IDC might replace verification measurements for indications with high passing rates.^20^ For this latter purpose, MC‐ or PB‐based algorithms are used in radiotherapy. However, these algorithms should be implemented independent from the dose calculation algorithm of the clinical TPS. A major drawback of IDC, however is that, it is not sensitive to failures of the beam delivery system, if it does not use log‐file information from the beam monitor system. For indications with highest failure rates (e.g., neck with 88%) when using IDC, further investigations by measurements are still considered necessary.

HIT is currently commissioning the myQA iON tool (IBA Dosimetry, Schwarzenbruck, Germany), which uses the MCsquare dose engine. This commercial tool is currently used for scanned protons and will be expanded to carbon ion beams.^35^, ^36^ Dreindl et al. reported that they reduced the experimental PSQA for protons initially by about 25 % and reached up to 90 % after 1 year with this tool.^36^ Moyers et al. reported that they reduced the number of PSQA measurements for subsets of patient groups that had similar field characteristics and high passing rates with their MC‐based fast dose calculations.^20^ On the other side, Matter et al. found that PSQA measurements were ineffective in detecting data transfer errors and suboptimal machine performance in dose, and that machine‐based QA and log file IDCs were capable of detecting workflow errors.^17^ These methods therefore provide a valuable and reliable enhancement to existing quality assurance protocols and could potentially replace measurements. Finally, it has to be noted that independent recalculation with independent analytical or Monte Carlo algorithms alone can capture only errors in the dose calculation of the TPS while measurements are additionally able to detect deviations in the beam delivery.

#### 4.4.2 A revised PSQA concept

When reducing dosimetric patient plan verifications, the question arises, whether additional measures are needed to ensure an accurate dose delivery within clinically defined tolerance limits. There are several risks for errors and failure modes in the clinical WF from treatment planning to dose delivery, which have to be detected or minimized prior to the first irradiation.^20^, ^41^ Robertson et al.^25^ reported that although log file‐based PSQA is more efficient and less risky than measurement‐based methods, both address different risks. They also reported that overall PSQA reduced risk most with log file‐based PSQA (39.2 %) and measurement‐based PSQA (31.8 %), whereas IDC contributed the least (6.8%). Combining methods improved reductions further: for instance, IDC with measurement‐ or log file‐based PSQA led to an average risk reduction of 36.7% or 45.3%, respectively, across the entire workflow, suggesting that combining the two methods is reasonable.

Based on our work, PSQA should include various checks to ensure an optimal patient safety and treatment quality: First, the integrity of data transfer between the treatment planning system and treatment delivery system can be validated using an in‐house tool, which should be validated. Second, even a highly accurate and extensive machine QA program can only keep the beam delivery within the institutional tolerance limits. Thus, international guidelines and external audits are recommended to assess the QA procedures by independent experts. Third, current delivery systems at particle therapy facilities are equipped with highly accurate and redundant monitoring systems, preventing wrong beam positions, spot sizes, and scan paths. Fourth, machine log file‐based analysis can be helpful since modern delivery systems offer beam log data in real time, which can be used to reconstruct and compare the delivered with the planned dose distribution on the actual patient CT, using the pencil beam characteristics recorded by the monitoring system.^21^, ^22^, ^23^, ^24^ Fifth, the IDC should be performed on the treatment planning CT or the geometry of the WP to verify the dose calculation of the clinical TPS, starting with small tolerances. In case, the IDC fails, a PSQA measurement should be triggered. Finally, for new types of treatment plans, measurements should always be performed. This may include using a new ion species or a new optimizing technique. In these cases, experience with PSQA measurements should be collected and only at later stages, the necessity of verification measurements might be discussed. To realize this, risk‐adapted decision‐making flow charts have to be developed, which can be used to determine the most appropriate PSQA verification measure.

## 5 CONCLUSION

Our results of dose verification measurements at HIT showed high passing rates across all PSQA measurements, reflecting the robustness of the comprehensive beam delivery QA program implemented at our institution. However, conventional PSQA remains time‐consuming, resource‐intensive, and is not compatible with online APT, which is increasingly being used in modern radiotherapy. As a result, there is a growing need to streamline or replace PSQA measurements by other methods such as IDC and/or machine‐ and log file‐based dose verification methods as it is already being done routinely for integrated adaptive therapy devices, like the MR‐Linac (Magnetic Resonance Linear Accelerator) or modern CBCT‐based systems (Cone Beam Computed Tomography), like the ETHOS. These approaches can help ensure reliable performance of the treatment delivery system, TPS, and clinical workflow, while also enabling detection of data transfer errors, an aspect that can additionally be monitored using independent in‐house tools. Nevertheless, novel or extreme treatment scenarios outside the established clinical experience should still undergo measurement‐based dose verification to confirm feasibility and dosimetric accuracy. To guide such decisions, a risk‐adapted PSQA protocol is recommended.

Future work will focus on investigating the dose outliers in the data, comparing IDC versus measurement‐based patient treatment plan verification, and developing a new PSQA strategy. This strategy will define when to use IDC, log file analysis, or dose measurements to optimize verification workflows while maintaining high treatment quality and safety.

## CONFLICT OF INTEREST STATEMENT

The authors declare no conflict of interest.

## Supporting information



Supporting Information

## Data Availability

The data supporting this study are not publicly available as they are still being used for ongoing research and future publications. Access may be granted upon reasonable request and subject to approval.

## References

[1] Suit H , DeLaney T , Goldberg S et al. Proton vs carbon ion beams in the definitive radiation treatment of cancer patients. Radiother Oncol. 2010;95(1):3‐22. doi:10.1016/j.radonc.2010.01.015 20185186

[2] Haberer T , Becher W , Schardt D , Kraft G . Magnetic scanning system for heavy‐ion therapy. (in English), Nucl Instrum Meth A. 1993;330(1–2):296‐305. doi:10.1016/0168-9002(93)91335-K

[3] Lomax AJ , Böhringer T , Bolsi A et al. Treatment planning and verification of proton therapy using spot scanning: initial experiences. Med Phys. 2004;31(11):3150‐3157. doi:10.1118/1.1779371 15587667

[4] Eichkorn T , König L , Held T et al. Carbon ion radiation therapy: one decade of research and clinical experience at heidelberg ion beam therapy center. Int J Radiat Oncol Biol Phys. 2021;111(3):597‐609. doi:10.1016/j.ijrobp.2021.05.131 34560023

[5] Karger CP , Peschke P . RBE and related modeling in carbon‐ion therapy. Phys Med Biol. 2017;63(1):01TR02. doi:10.1088/1361-6560/aa9102 28976361

[6] Jäkel O , Hartmann GH , Karger CP , Heeg P , Rassow J . Quality assurance for a treatment planning system in scanned ion beam therapy. Med Phys. 2000;27(7):1588‐1600. doi:10.1118/1.599025 10947262

[7] ICRU , “Prescribing, recording, and reporting light ion beam therapy,” Oxford University, Oxford University Press, ICRU Report 93, 2019, vol. 16 issue 1‐2.

[8] Flanz J , Litzenberg D , Chen H , Schreuder N . PTCOG white paper on particle therapy efficiency: aspects of quality assurance, verion 1 (Particle Therapy Co‐Operative Group, Boston). Newsletter for the Particle Therapy Co‐Operative Group (PTCOG), White Paper 2016. [Online]. Available: https://ptcog.site/images/Docs/White%20Paper%20QA%20Efficiency_Final.pdf

[9] Karger CP , Jäkel O , Palmans H , Kanai T . Dosimetry for ion beam radiotherapy. Phys Med Biol. 2010;55(21):R193‐234. doi:10.1088/0031-9155/55/21/R01 20952816

[10] Zhu XR , Poenisch F , Song X et al. Patient‐specific quality assurance for prostate cancer patients receiving spot scanning proton therapy using single‐field uniform dose. Int J Radiat Oncol Biol Phys. 2011;81(2):552‐559. doi:10.1016/j.ijrobp.2010.11.071 21300457

[11] Trnková P , Bolsi A , Albertini F , Weber DC , Lomax AJ . Factors influencing the performance of patient specific quality assurance for pencil beam scanning IMPT fields. Med Phys. 2016;43(11):5998. doi:10.1118/1.4964449 27806620

[12] Martišíková M , Brons S , Hesse BM , Jäkel O . High‐resolution fluence verification for treatment plan specific QA in ion beam radiotherapy. Phys Med Biol. 2013;58(6):1725‐1738. doi:10.1088/0031-9155/58/6/1725 23429210

[13] Karger CP , Jäkel O , Hartmann GH , Heeg P . A system for three‐dimensional dosimetric verification of treatment plans in intensity‐modulated radiotherapy with heavy ions. Med Phys. 1999;26(10):2125‐2132. doi:10.1118/1.598728 10535629

[14] Henkner K , Winter M , Echner G et al. A motorized solid‐state phantom for patient‐specific dose verification in ion beam radiotherapy. Phys Med Biol. 2015;60(18):7151‐7163. doi:10.1088/0031-9155/60/18/7151 26334387

[15] Mirandola A , Molinelli S , Vilches Freixas G et al. Dosimetric commissioning and quality assurance of scanned ion beams at the Italian National Center for Oncological Hadrontherapy. Med Phys. 2015;42(9):5287‐5300. doi:10.1118/1.4928397 26328978

[16] Arjomandy B , Sahoo N , Ciangaru G , Zhu R , Song X , Gillin M . Verification of patient‐specific dose distributions in proton therapy using a commercial two‐dimensional ion chamber array. Med Phys. 2010;37(11):5831‐5837. doi:10.1118/1.3505011 21158295

[17] Matter M , Nenoff L , Meier G , Weber DC , Lomax AJ , Albertini F . Alternatives to patient specific verification measurements in proton therapy: a comparative experimental study with intentional errors. Phys Med Biol. 2018;63(20):205014. doi:10.1088/1361-6560/aae2f4 30234498

[18] Meier G , Besson R , Nanz A , Safai S , Lomax AJ . Independent dose calculations for commissioning, quality assurance and dose reconstruction of PBS proton therapy. Phys Med Biol. 2015;60(7):2819‐2836. doi:10.1088/0031-9155/60/7/2819 25779992

[19] Meijers A , Guterres Marmitt G , Ng Wei Siang K et al. Feasibility of patient specific quality assurance for proton therapy based on independent dose calculation and predicted outcomes. Radiother Oncol. 2020;150:136‐141. doi:10.1016/j.radonc.2020.06.027 32579999

[20] Moyers MF , Wang Q , Deng Y et al. Verification of an independent dose calculation method for portal‐specific QA of proton and carbon ion beams. Radiation Medicine and Protection 2022;3(3):152‐157. doi:10.1016/j.radmp.2022.05.004

[21] Li H , Sahoo N , Poenisch F et al. Use of treatment log files in spot scanning proton therapy as part of patient‐specific quality assurance. Med Phys 2013;40(2):021703. doi:10.1118/1.4773312 23387726 PMC3555925

[22] Meijers A , Jakobi A , Stützer K et al. Log file‐based dose reconstruction and accumulation for 4D adaptive pencil beam scanned proton therapy in a clinical treatment planning system: implementation and proof‐of‐concept. Med Phys. 2019;46(3):1140‐1149. doi:10.1002/mp.13371 30609061

[23] Scandurra D , Albertini F , van der Meer R et al. Assessing the quality of proton PBS treatment delivery using machine log files: comprehensive analysis of clinical treatments delivered at PSI Gantry 2. Phys Med Biol. 2016;61(3):1171‐1181. doi:10.1088/0031-9155/61/3/1171 26767316

[24] Yun Y , Han MC , Kim C et al. Enhancing patient‐specific quality assurance in carbon‐ion radiation therapy: recalculating delivered dose distribution using log data. In Vivo. 2025;39(2):1086‐1093. doi:10.21873/invivo.13913 40010991 PMC11884496

[25] Robertson D et al. PTCOG White Paper on Risk Assessment of Patient‐Specific Quality Assurance (PSQA) Methods in Particle Therapy (accessed 29 July 2025). Newsletter for the Particle Therapy Co‐Operative Group (PTCOG), White Paper (summary) 2024. [Online]. Available: https://www.ptcog.site/index.php/other‐subcommittees?view=article&id=97&catid=10

[26] Haberer T , Debus J , Eickhoff H , Jäkel O , Schulz‐Ertner D , Weber U . The heidelberg ion therapy center. Radiother Oncol. 2004;73(Suppl 2):S186‐90. doi:10.1016/s0167-8140(04)80046-x 15971340

[27] Combs SE , Jäkel O , Haberer T , Debus J . Particle therapy at the Heidelberg Ion Therapy Center (HIT)—Integrated research‐driven university‐hospital‐based radiation oncology service in Heidelberg, Germany. Radiother Oncol. 2010;95(1):41‐44. doi:10.1016/j.radonc.2010.02.016 20227124

[28] Jäkel O . Medical physics aspects of particle therapy. Radiat Prot Dosimetry. 2009;137(1–2):156‐166. doi:10.1093/rpd/ncp192 19828718

[29] Tessonnier T , Ecker S , Besuglow J et al. Commissioning of helium ion therapy and the first patient treatment with active beam delivery. Int J Radiat Oncol Biol Phys. 2023;116(4):935‐948. doi:10.1016/j.ijrobp.2023.01.015 36681200

[30] Carlino A , Stock M , Zagler N et al. Characterization of PTW‐31015 PinPoint ionization chambers in photon and proton beams. Phys Med Biol. 2018;63(18):185020. doi:10.1088/1361-6560/aadd39 30152791

[31] INTERNATIONAL ATOMIC ENERGY , Absorbed Dose Determination in External Beam Radiotherapy. International Atomic Energy Agency; 2001.

[32] Zhu X , Li Y , Mackin D et al. Towards effective and efficient patient‐specific quality assurance for spot scanning proton therapy. Cancers (Basel). 2015;7(2):631‐647. doi:10.3390/cancers7020631 25867000 PMC4491675

[33] Mackin D , Zhu XR , Poenisch F et al. Spot‐scanning proton therapy patient‐specific quality assurance: results from 309 treatment plans. International Journal of Particle Therapy. 2014;1(3):711‐720. doi:10.14338/IJPT-14-00017.1

[34] Noel CE , Santanam L , Parikh PJ , Mutic S . Process‐based quality management for clinical implementation of adaptive radiotherapy. Med Phys. 2014;41(8):081717. doi:10.1118/1.4890589 25086527 PMC4119199

[35] Cohilis M , Hong L , Janssens G et al. Development and validation of an automatic commissioning tool for the Monte Carlo dose engine in myQA iON. Phys Med. 2022;95:1‐8. doi:10.1016/j.ejmp.2022.01.002 35051680

[36] Dreindl R , Bolsa‐Ferruz M , Fayos‐Sola R et al. Commissioning and clinical implementation of an independent dose calculation system for scanned proton beams. J Appl Clin Med Phys. 2024;25(5):e14328. doi:10.1002/acm2.14328 38553788 PMC11087175

[37] Grevillot L , Boersma DJ , Fuchs H et al. Technical Note: gATE‐RTion: a GATE/Geant4 release for clinical applications in scanned ion beam therapy. Med Phys. 2020;47(8):3675‐3681. doi:10.1002/mp.14242 32422684

[38] Kozłowska WS , Böhlen TT , Cuccagna C et al. FLUKA particle therapy tool for Monte Carlo independent calculation of scanned proton and carbon ion beam therapy. Phys Med Biol. 2019;64(7):075012. doi:10.1088/1361-6560/ab02cb 30695766

[39] Chen Z , Moyers M , Deng Y et al. Analysis of delivery and recalculation of dose using DICOM treatment records. (in English), Radiation Medicine and Protection, A R T I C L E I N F O. 2022;3(3):123‐130. doi:10.1016/j.radmp.2022.06.002

[40] Kry SF , Molineu A , Kerns JR et al. Institutional patient‐specific IMRT QA does not predict unacceptable plan delivery. Int J Radiat Oncol Biol Phys. 2014;90(5):1195‐1201. doi:10.1016/j.ijrobp.2014.08.334 25442044 PMC4276500

[41] O'Daniel J , Hernandez V , Clark C et al. Which failures do patient‐specific quality assurance systems need to catch?. Med Phys. 2025;52(1):88‐98. doi:10.1002/mp.17468 39466302

[42] Huq MS , Fraass BA , Dunscombe PB et al. The report of Task Group 100 of the AAPM: application of risk analysis methods to radiation therapy quality management. Med Phys. 2016;43(7):4209. doi:10.1118/1.4947547 27370140 PMC4985013

[43] Klein EE , Hanley J , Bayouth J et al. Task Group 142 report: quality assurance of medical accelerators. Med Phys. 2009;36(9):4197‐4212. doi:10.1118/1.3190392 19810494

[44] Hanley J , Dresser S , Simon W et al. AAPM Task Group 198 Report: an implementation guide for TG 142 quality assurance of medical accelerators. Med Phys. 2021;48(10):e830‐e885. doi:10.1002/mp.14992 34036590

[45] Baltazar F , Tessonnier T , Mein S et al. Development and dosimetric verification of static SHArc: step‐and‐shoot carbon ion arc therapy for LET(d) escalation in pancreatic tumors. Med Phys. 2025;52(10):e70055. doi:10.1002/mp.70055 41058526 PMC12505198

[46] Albertini F , Czerska K , Vazquez M et al. First clinical implementation of a highly efficient daily online adapted proton therapy (DAPT) workflow. Phys Med Biol. 2024;69(21):215030, doi:10.1088/1361-6560/ad7cbd 39293489

[47] Lima TV , Dosanjh M , Ferrari A , Molineli S , Ciocca M , Mairani A . Monte Carlo calculations supporting patient plan verification in proton therapy. Front Oncol 2016;6:62, doi:10.3389/fonc.2016.00062 27047796 PMC4796019

[48] Magro G , Molinelli S , Mairani A et al. Dosimetric accuracy of a treatment planning system for actively scanned proton beams and small target volumes: monte Carlo and experimental validation. Phys Med Biol. 2015;60(17):6865‐6880. doi:10.1088/0031-9155/60/17/6865 26301623

[49] Miften M , Olch A , Mihailidis D et al. Tolerance limits and methodologies for IMRT measurement‐based verification QA: recommendations of AAPM Task Group No. 218. Med Phys. 2018;45(4):e53‐e83. doi:10.1002/mp.12810 29443390

[50] Vidal M , Stolarczyk L . Towards a consensus on pre treatment verification in particle therapy. European Society for Radiotherapy and Oncology (ESTRO). https://www.estro.org/Workshops/Past‐Workshop/2023/2023‐Physics‐Workshop‐Science‐in‐Development/Towards‐a‐consensus‐on‐pre‐treatment‐verification (accessed 29 July 2025)

