# Supplementary material for: Patient‐specific quality assurance at the Heidelberg Ion Beam Therapy Center: 10 years experience in treatment plan verification
Source: Med Phys. 2025 Dec 26;53(1):e70237. doi: 10.1002/mp.70237 (PMC12742551; doi:10.1002/mp.70237)
Supplement: Supplementary file 1 — Supporting Information [file MP-53-0-s001.docx]

TABLE S 1: Number of individual measurement points (activated and deactivated ICs) and number of measurements complying with certain tolerances.

| **Year** | **Number of measured points** |  | **Activated ICs with dose deviations within** | | | | | **Deactivated ICs with dose deviations within** |
| --- | --- | --- | --- | --- | --- | --- | --- | --- |
|  |  | **Activated ICs (%)** | **±5%** | **±7%** | **±10%** | **±15%** | **±20%** | **±7 (%)** |
| **2016** | 54883 | 83 | 95.41% | 98.56% | 99.69% | 99.95% | 100% | 14.0 |
| **2017** | 50316 | 84 | 95.91% | 98.73% | 99.81% | 99.99% | 100% | 14.3 |
| **2018** | 63008 | 83 | 97.00% | 99.14% | 99.85% | 99.99% | 100% | 15.6 |
| **2019** | 48642 | 83 | 97.86% | 99.50% | 99.88% | 99.98% | 100% | 15.7 |
| **2020** | 59998 | 82 | 97.61% | 99.31% | 99.83% | 99.97% | 100% | 16.1 |
| **2021** | 56808 | 84 | 98.33% | 99.62% | 99.92% | 99.99% | 100% | 14.8 |
| **2022** | 64227 | 84 | 98.03% | 99.61% | 99.97% | 99.99% | 100% | 15.1 |
| **2023** | 57248 | 85 | 98.38% | 99.63% | 99.97% | 100% | 100% | 14.1 |
| **2024** | 59311 | 85 | 96.77% | 99.09% | 99.83% | 99.97% | 100% | 13.2 |
| **2025 (01-06)** | 36909 | 84 | 97.48% | 99.57% | 99.93% | 100% | 100% | 14.3 |
| **Total** | 551350 | 84±0.9 | 97.28±1.00% | 99.28±0.40% | 99.87±0.09% | 99.98±0.02% | 100% | 15±0.9 |

Abbreviations: IC = Ionization chamber, SD = standard deviation.

TABLE S 2: Summary of repeated measurements. Number of measurements and mean ± SD of the deviations for the original and repeated measurements of the same irradiation plans. No statistically significant differences were found between the original and repeated measurements (t-test, p > 0.05).

| **Year** | **Original Measurements** | | | **Repeated Measurements** | | |
| --- | --- | --- | --- | --- | --- | --- |
|  | **Number of**  **measurements** | **Mean±SD deviation [%]** | **P value**  **(t-test)** | **Number of**  **measurements** | **Mean±SD deviation [%]** | **P value**  **(t-test)** |
| **2016** | 89 | -1.2±1.6 | P < 0.01 | 102 | -1.4±1.0 | P < 0.01 |
| **2017** | 78 | -1.2±1.8 | P < 0.01 | 88 | -1.2±1.0 | P < 0.01 |
| **2018** | 61 | -0.9±1.6 | P < 0.01 | 63 | -1.1±1.4 | P < 0.01 |
| **2019** | 45 | -1.4±1.9 | P < 0.01 | 55 | -0.9±1.1 | P < 0.01 |
| **2020** | 68 | -0.6±2.4 | P < 0.01 | 77 | -0.8±1.1 | P < 0.01 |
| **2021** | 40 | -1.8±1.3 | P < 0.01 | 42 | -1.4±1.0 | P < 0.01 |
| **2022** | 39 | -0.9±1.1 | P < 0.01 | 43 | -0.9±1.1 | P < 0.01 |
| **2023** | 68 | -0.9±2.0 | P < 0.01 | 76 | -0.7±1.0 | P < 0.01 |
| **2024** | 69 | -0.04±4.5 | 0.01 < P < 0.05 | 70 | -0.3±1.2 | P > 0.05 |
| **2025 (01-06)** | 7 | -0.4±2.1 | P > 0.05 | 9 | -0.4±2.1 | P > 0.05 |
| **Total** | 564 | -0.9±0.5 | P < 0.01 | 625 | -1.0±0.4 | P < 0.01 |

Abbreviations: IC = ionization chamber, SD = standard deviation.
